# *Panx1* channels promote both anti- and pro-seizure-like activities in the zebrafish via *p2rx7* receptors and ATP signaling

**DOI:** 10.1038/s42003-022-03356-2

**Published:** 2022-05-18

**Authors:** Paige Whyte-Fagundes, Daria Taskina, Nickie Safarian, Christiane Zoidl, Peter L. Carlen, Logan W. Donaldson, Georg R. Zoidl

**Affiliations:** 1 Department of Biology, York University, Toronto, ON M3J1P3 Canada; 2 Center of Vision Research (CVR), York University, Toronto, ON M3J1P3 Canada; 3 Krembil Research Institute, University Health Network, 60 Leonard Ave, Toronto, ON M5T 1M8 Canada; 4 Department of Medicine, Physiology and BME, University of Toronto, 399 Bathurst St., 5w442, Toronto, ON M5T 2S8 Canada

**Keywords:** Molecular neuroscience, Epilepsy

## Abstract

The molecular mechanisms of excitation/inhibition imbalances promoting seizure generation in epilepsy patients are not fully understood. Evidence suggests that Pannexin1 (*Panx1*), an ATP release channel, modulates the excitability of the brain. In this report, we performed electrophysiological, behavioral, and molecular phenotyping experiments on zebrafish larvae bearing genetic or pharmacological knockouts of Panx1a and Panx1b channels, each homologous to human PANX1. When Panx1a function is lost, or both channels are under pharmacological blockade, seizures with ictal-like events and seizure-like locomotion are reduced in the presence of pentylenetetrazol. Transcriptome profiling by RNA-seq demonstrates a spectrum of distinct metabolic and cell signaling states which correlate with the loss of Panx1a. Furthermore, the pro- and anticonvulsant activities of both Panx1 channels affect ATP release and involve the purinergic receptor P2rx7. Our findings suggest a subfunctionalization of Panx1 enabling dual roles in seizures, providing a unique and comprehensive perspective to understanding seizure mechanisms in the context of this channel.

## Abstract (web summary)

The Panx1a and Panx1b (pannexin) ATP release channels exhibit pro- and anti-convulsant effects, respectively, in a zebrafish model, providing further insight into the role of pannexins in seizure development.

## Introduction

The widely accepted excitation/inhibition (E/I) imbalance theory of epileptic seizures places the imbalance of neuronal transmitters glutamate and GABA release first, but evidence is accumulating that this concept warrants reconsideration and expansion to account for genetic mutations with little effect on E/I balance, neurotransmitters which exert paradoxical effects, anti-seizure medications which do not necessarily work by decreasing excitation or increasing inhibition, and metabolic factors participating in the regulation of neuronal and network excitability^1^. Further, we also must account for altered ATP- and adenosine-mediated signaling between neurons and glial cells contributing to heightened states of excitability and epileptic seizures^2^. ATP release and signaling can be excitatory and inhibitory, but adenosine strongly inhibits electrical activity. Five groups of ATP-release channels with expression in the nervous system are known: pannexin-1 (Panx1)^3^, connexin hemichannels^4^, calcium homeostasis modulator 1^5^, volume-regulated anion channels, also known as volume-sensitive outwardly rectifying^6^, and maxi-anion channels^7^. Panx1 is recognized as a pro-convulsant channel after behavioral and electrophysiological markers of excitability are ameliorated in distinct models of epilepsy once Panx1 is inhibited pharmacologically or by global deletion in mice^8–11^. Other evidence for pro-convulsant actions of Panx1 derived from increased expression of human and rodent Panx1 found in epileptic tissue^9,12–14^. However, the simplistic view of inhibiting mammalian Panx1 and causing anticonvulsant effects is challenged. The targeted deletion of mouse Panx1 in astrocytes potentiates, while the absence of Panx1 in neurons attenuates seizure manifestation^15^. Furthermore, the contribution of Panx1 to seizures is also brain region-dependent^16^, advocating for cell-type specific pharmacological intervention.

In this study, we used zebrafish as a model to probe how the evolutionary divergence of mammalian and fish Panx1 channels affects susceptibility to epileptic seizures. Fish and mammals are separated by about 400–600 million years of biological evolution. During that time, *Panx2* and then *Panx1* and *Panx3* genes originated from a common ancestor as part of two rounds of genome duplication that occurred at the early stage of vertebrate divergence^17,18^. *Panx1a* and *panx1b* genes most likely originated from a third major genome duplication event in teleost fish, about 320–350 million years ago^18–20^. Evolutionary conservation implies that epilepsy can be investigated in zebrafish^21–24^. Models of Dravet Syndrome and 40 single-gene mutant zebrafish lines representing childhood epilepsies exemplify the generation of models by mutagenic screens or gene-targeting^25,26^. Further, zebrafish-centric drug discovery has brought antiepileptic drugs to clinical studies and the bedside^27–29^.

The two zebrafish ohnologs, *panx1a* and *panx1b* have distinct expression localization and biophysical properties^19,20,30–33^. Like mammalian Panx1^18,34,35^, the zebrafish *panx1a* is broadly expressed in all tissues tested^20,30^, whereas the expression of *panx1b* is highest in the nervous system^20^. We suggested that both pannexins fulfill different functions in vivo based on differences in the unitary conductance of Panx1a (≈380pS) and Panx1b (480–500pS) channels, the complexity of subconductance stages, and the cumulative open and closed times^20^. Here, we interrogated Panx1 channels genetically and pharmacologically, and induced seizure-like activities in the zebrafish using pentylenetetrazole (PTZ)^36^ to suppress inhibitory networks by blocking gamma-aminobutyric acid (GABA)-A receptors. The zebrafish responses to PTZ were physiologically and behaviorally comparable to mammals^36,37^.

Gene-edited zebrafish lines lacking Panx1a or Panx1b protein expression were generated in-house^32,38,39^. The fish behave normally and do not seize spontaneously—treatment with PTZ induced opposite seizure-like phenotypes and morbidity. Targeted ablation of *panx1b* potentiates, while the absence of *panx1a* attenuates seizure manifestations according to recordings of in vivo local field potentials (LFP) and locomotor behavior. Deletion of both fish *panx1* genes in a double knockout fish (DKO) causes a moderate phenotype with reduced seizures. In line with these observations are significant changes to extracellular ATP levels. The acute pharmacological blocking of both Panx1 channels using Probenecid (PROB) abolished PTZ-induced seizures. The molecular, electrophysiological, and behavioral changes overlap but are not identical to genetic interference. Likewise, pharmacological blocking of the Panx1 interaction partner P2rx7^40^ reduces seizure-like activities like PROB treatment. Finally, structural modeling and comparing the pores of the two Panx1 ohnologs and the human PANX1 channel supports our experimental data implicating the Panx1a protein as a driver of pro-convulsant activities.

Our analysis of genetic and pharmacological models in the zebrafish establishes Panx1a channels as the pro-convulsant Panx1 channel. The different propensities of Panx1a and Panx1b channels in developing seizure-like activities can take us a step closer to understanding seizure mechanisms and prompt drug discovery initiatives by targeting shared properties of zebrafish and humans PANX1.

## Results

### Panx1 genotypes determine evoked seizure-like events

**Fig. 1:**
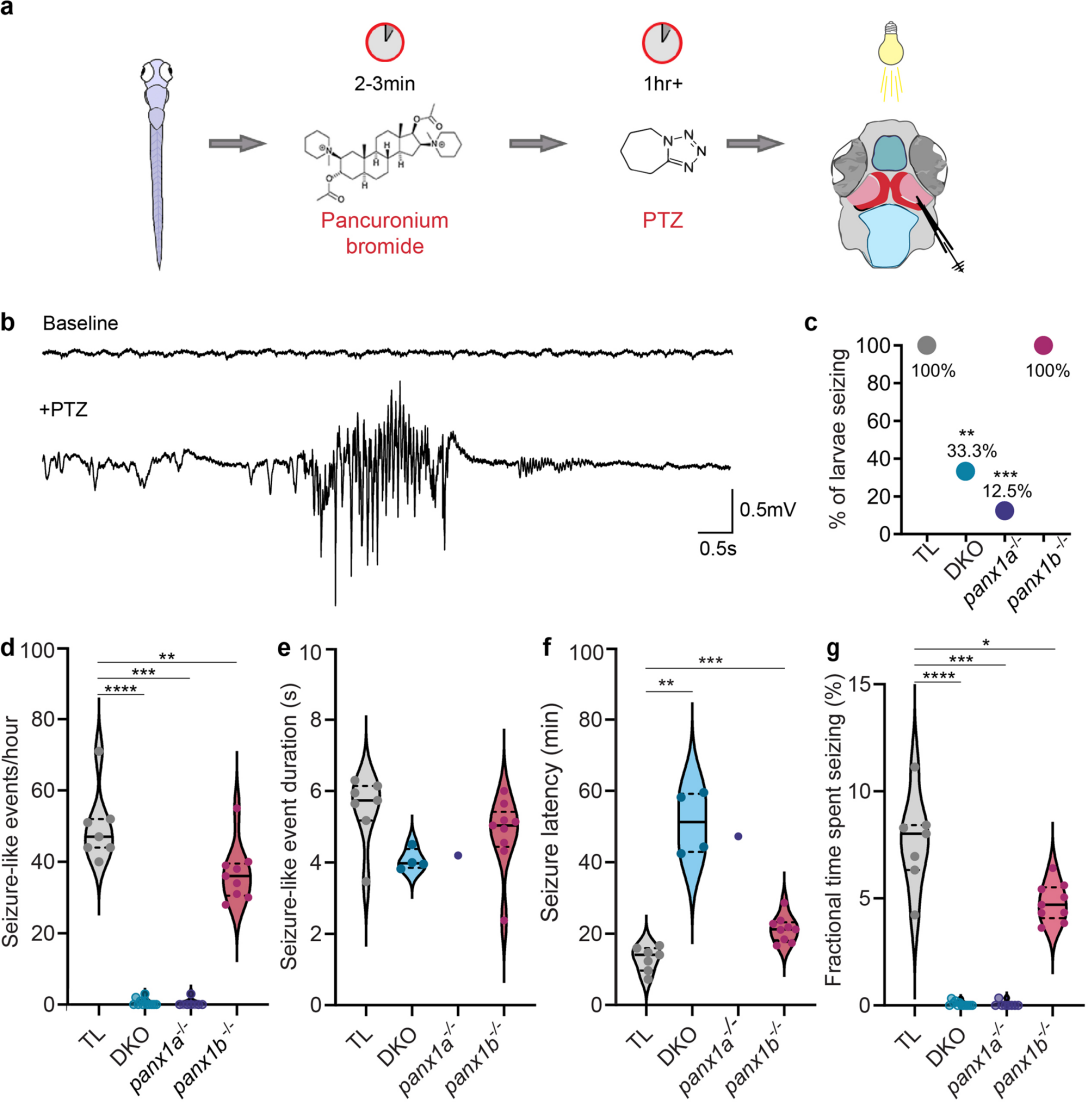
*Panx1* knockout larvae showed distinct seizure-like activities in vivo. **a** Workflow for recording in vivo local field potentials (LFP) from the right optic tectum (OT) of 7dpf anesthetized larva after 15 mM PTZ treatment. **b** Representative recording of baseline activity and a seizure-like event (SLE) induced with the addition of PTZ from a TL larva. **c** All TL and *panx1b*^−*/*−^ larvae had SLEs (TL (gray) *n* = 7/7; DKO (light blue) *n* = 4/12, *P* = 4.5 × 10^−3^; *panx1a*^−*/*−^ (deep blue) *n* = 1/8, *P* = 3.0 × 10^−4^; *panx1b*^−*/*−^ (magenta) *n* = 9/9, *P* = 1.0, Barnard test). **d** Quantification of SLEs in the first hour of LFP recording revealed that all *panx1* knockout (KO) larvae had a significant reduction in SLEs compared to PTZ treated TL controls, presented as an average number of SLEs/hour ± s.e.m. (DKO: *P* = < 1.0 × 10^−4^; *panx1a*^*−/*−^: *P* = 3.0 × 10^−4^; *panx1b*^*−/*−^*P* = 5.7 × 10^−3^). **e** The average duration of SLEs (in seconds ± s.e.m.) for each genotype was not significantly different compared to TLs. Statistical tests were not significant for *panx1a*^−*/*−^ due to lack of statistical power. (DKO: *P* = 0.07; *panx1b*^−*/*−^: *P* = 0.09). **f** All *panx1* knockout larvae had a significant delay in the average onset time (minutes ± s.e.m.) of the first seizure-like event compared to PTZ treated TL controls. (DKO: *P* = 6.1 × 10^−3^; *panx1b*^−*/*−^: *P* = 3.0 × 10^−4^). **g** All *panx1* knockout larvae spent significantly less time seizing compared to PTZ treated TL controls. Average fractional time spent seizing is presented in percent ± s.e.m. (DKO: *P* = < 1.0 × 10^−4^; *panx1a*^−*/*−^: *P* = 3.0 × 10^−4^; *panx1b*^−*/*−^: *P* = 0.01). *N* = number of larvae. Statistical Mann–Whitney U test in (**d–g**). Scale bars 0.5 mV by 0.5 s. *P < 0.05, **P < 0.01, ***P < 0.001, ****P < 0.0001.

Seizure-like events (SLE) were recorded from the optic tectum (OT) of 7days post fertilization (dpf) larvae as described by^41^ (Fig. 1a). In the OT, the Panx1a protein was primarily found in the neuropil of arborization fields (AFs) (Supplementary Fig. 1, a, a’). The Panx1b protein was detected in densely compacted nuclear layers of the OT (Supplementary Fig. 1, b, b’). The localization of both Panx1a and Panx1b in the OT suggested expression in neurons. Astrocytes in the OT are GFAP-negative, and identification of Panx1a or Panx1b was not possible due to a lack of a definitive biomarker. Panx1a or Panx1b was not found in GFAP-positive radial glial cells in the spinal cord (Supplementary Fig. 1c–e).

Characteristic SLEs induced by 15 mM PTZ treatment were high-frequency events that appeared as initial large bursts followed by short paroxysmal bursts absent in baseline recordings (Fig. 1b**;** Supplementary Fig. 2). The number of *panx1a*^*−/−*^ larvae exhibiting SLEs (*n* = 1; 8 tested) was reduced by 88% compared to PTZ treated Tubingen Longfin (TL) controls (*n* = 7; 7 tested). All *panx1b*^*−/−*^ larvae (*n* = 9; 9 tested) and 30% of the DKO larvae seized (DKO*;* *n* = 4; 12 tested). SLEs in DKOs were significantly reduced compared to controls and indistinguishable from *panx1a*^*−/−*^ larvae (Fig. 1c).

The average number of SLEs per hour was similar for DKOs and *panx1a*^−/−^ larvae (*P* = 0.58), with less than one event on average (DKO: 0.58 ± 0.29; *panx1a*^*−/−*^: 0.38 ± 0.38 events/hour). Even though all *panx1b*^*−/*−^ fish had SLEs, the average number of events per hour were significantly less compared to controls (*panx1b*^−*/*−^*:* 36.78 ± 2.67; TL: 49.86 ± 3.86 events/hour, *P* = 0.006) (Fig. 1d). The duration of events in all genotypes were comparable to TL controls (TL: 5.5 s ± 0.4 s; DKO: 4.1 s ± 0.2 s, *P* = 0.07; *panx1a*^−/−^: 4.2 s, *P* = n/a; *panx1b*^*−/−*^: 4.8 s ± 0.4 s, *P* = 0.09) (Fig. 1e).

The latency until the first SLE showed differences amongst genotypes. SLEs started in TL after 12.9 ± 1.3 min. *Panx1b*^*−/*−^ larvae followed at 21 ± 1.2 min (*P* = 0.0003). DKO (51.1 ± 4.5 min, *P* = 0.006) and *panx1a*^−/−^ larvae responded last to PTZ treatment (47.3 min, *P* = n/a) (Fig. 1f). Although *panx1a*^−*/−*^ larvae were last to respond to PTZ treatment, neither the event duration nor the latency was significantly different from TLs. Only one larva exhibited a single SLE, which caused a lack of statistical power in the data analysis. The fractional percentage of seizing time within the first hour was reduced in *panx1b*^−*/−*^ larvae compared to TL controls (*panx1b* ^−*/−*^: 4.8% ± 0.3%; TL: 7.6% ± 0.8%). Both DKO and *panx1a*^*−/*−^ larvae displayed minimal seizing time (<0.1%). (Fig. 1g).

### Loss of Panx1 caused local network differences in the optic tectum of PTZ treated larvae

**Fig. 2:**
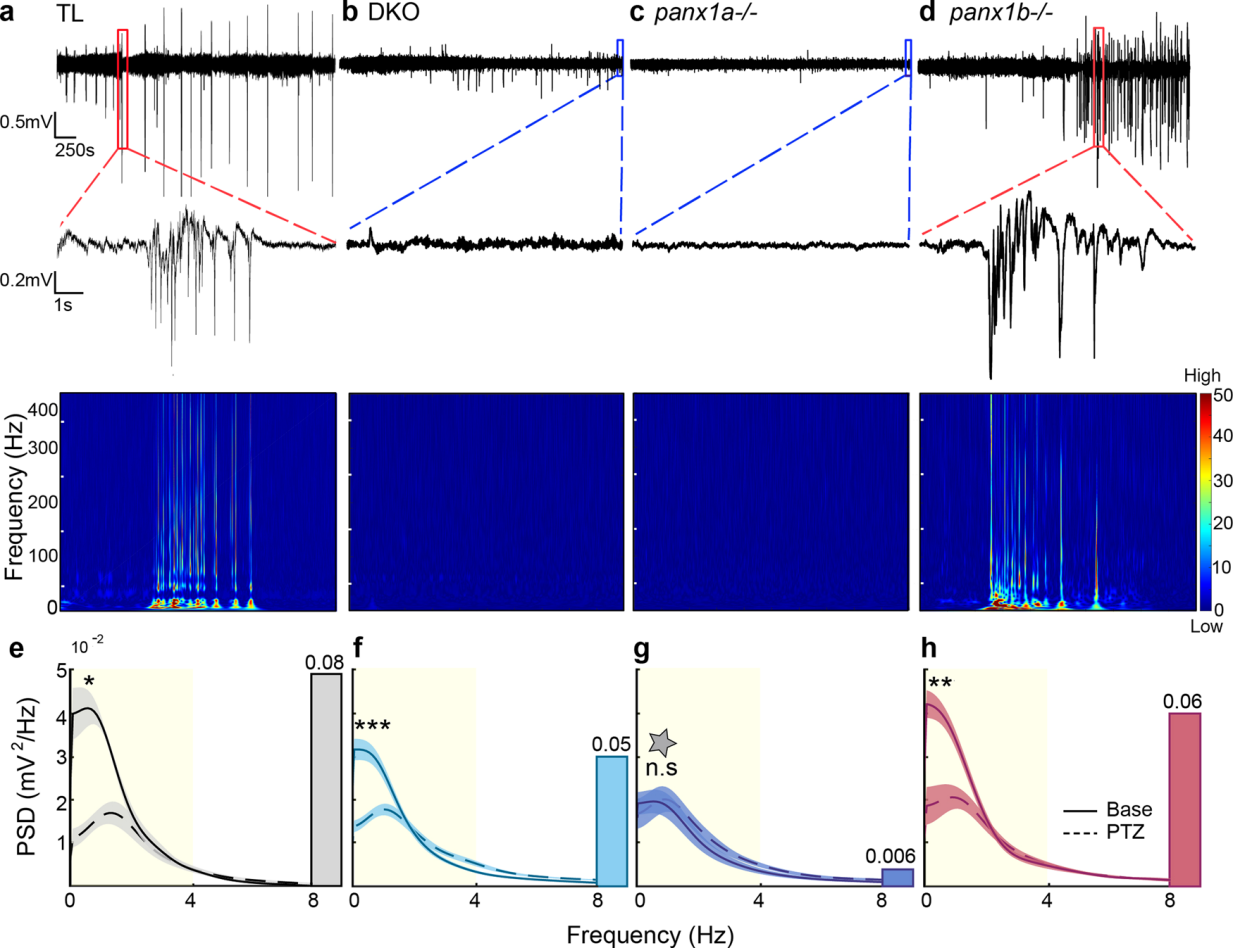
Spectral Analysis of LFPs revealed unique differences in larvae. Representative 1 h long LFP traces with PTZ treatment: **a** TL (**b**) DKO, (**c**) *panx1a*^−*/*−^, and (**d**) *panx1b*^−*/*−^. TL and *panx1b*^−*/−*^ traces showed typical seizure-like events. Single events were highlighted by red dotted lines and magnified. Blue dotted lines highlight a lack of seizure-like activity in DKO and *panx1a*^−*/*−^ larvae. Spectrograms below the traces showed low and high-frequency power increases during seizure-like events for TL and *panx1b*^−*/*−^. Scale bar: A value of 0 (blue) is low, and a value of 50 (red) is high. **e–h** Power spectral density (PSD, mV^2^/Hz) measured across frequencies revealed no significant differences in baseline (dotted lines) frequencies for all genotypes (*P* = 0.69; Kruskal–Wallis test). **e** TL, gray; **f** DKO, light blue; **g** *panx1a*^*−/−*^, dark blue; **h** *panx1b*^−*/*−^, magenta. The power was significantly increased in the delta band (1–4 Hz) after PTZ treatment (solid line) for all genotypes except *panx1a*^*−/*−^ (TL: *P* = 0.02, *n* = 7; DKO: *P* = 5.0 × 10^−4^, *n* = 12; *panx1a*^−*/*−^: *P* = 0.6, *n* = 8; *panx1b*^−*/*−^: *P* = 3.9 × 10^−3^, *n* = 9; Wilcoxon signed-rank test). The PSD was plotted as an average across traces with the shaded regions indicating ± s.e.m. Changes in delta power were quantified from the areas under the curves (AUC; highlighted in yellow) of the power spectrum. The changes in AUCs are represented as bar graphs inserted to the right of the power spectra. TL showed the greatest change in delta, followed by *panx1b*^*−/*−^ (*P* = 1.0), and DKO (*P* = 0.4). *Panx1a*^−*/*−^ exhibited insignificant changes (*P* = 2.3 × 10^−3^; Dunn’s multiple comparisons) and maintained delta power throughout recordings. *N* = number of larvae. Scale bars: top = 0.5 mV by 250 s and bottom = 0.2 mV by 1 s. ***P < 0.001, ****P < 0.0001, gray star - *P* = 2.3 × 10^−3^ compared to TL with PTZ.

The time-frequency domains defined PTZ induced SLEs across genotypes. Representative 1 h traces of electrographic recordings for TL controls, DKO, *panx1a*^*−/*−^, and *panx1b*^−*/*–^ larvae after PTZ application are shown in Fig. 2a–d. The exposure to PTZ elicited discharges characterized by large amplitudes and poly-spiking activities of at least 3 s in duration. TL controls and *panx1b*^*−/*−^ larvae showed typical SLEs (red dotted lines zoom into these regions). Highlighted events near the end of the representative traces for DKO and *panx1a*^*−/*−^ larvae demonstrated the lack of seizure-like electrographic signatures (blue dotted lines zoom into these regions). LFPs revealed changes in spectral power in the time and frequency domains visible in expanded views of the corresponding spectrograms. TL controls (**2a**) and *panx1b*^−/−^ larvae (**2d**) demonstrated a robust increase in low-frequency power and increased power in high frequencies above 60 Hz. The spectrograms for DKO (**2b**) and *panx1a*^−*/*−^ larvae (**2c**) revealed frequency power like baseline activity when scaled to match TL (**2a**) and *panx1b*^*−/*−^ (**2b**) data (Supplementary Fig. 3).

The power spectral density was measured to identify tectal network differences. Baseline activities were similar across genotypes (*P* = 0.69 for each comparison). Changes were seen in delta power (highlighted in yellow) after PTZ treatment and quantified by measuring the difference in the area under the power spectrum from 1 to 4 Hz (inset bar graphs) (Fig. 2 e–h). PTZ treatment significantly increased delta power for TL (**2e**), DKO (**2f)**, and *panx1b*^−*/*−^ larvae (**2h**). In contrast, delta power for *panx1a*^*−/−*^ remained at a base level (**2g**; dark blue bar) or was significantly decreased compared to PTZ treated TL controls (**2g;** gray star, *P* = 0.0023). *Panx1b*^*−/−*^ (*P* = 0.99) and DKO larvae (*P* = 0.4) showed similar delta power during PTZ treatment compared to TL (TL = 0.08 (gray bar), *panx1b*^−/−^ = 0.06 (magenta bar), and DKO = 0.05 (light blue bar)). The electrical discharges for both TL controls and *panx1b*^−*/−*^ larvae were similar in waveform to those previously reported in zebrafish^36,41^. However, they occurred less frequently in *panx1b*^*−/*−^ and were absent in *panx1a*^*−/−*^ larvae.

#### *DKO and panx1a*^*−/−*^*larvae* have reduced interictal-like epileptiform discharge activity in the optic tectum

**Fig. 3:**
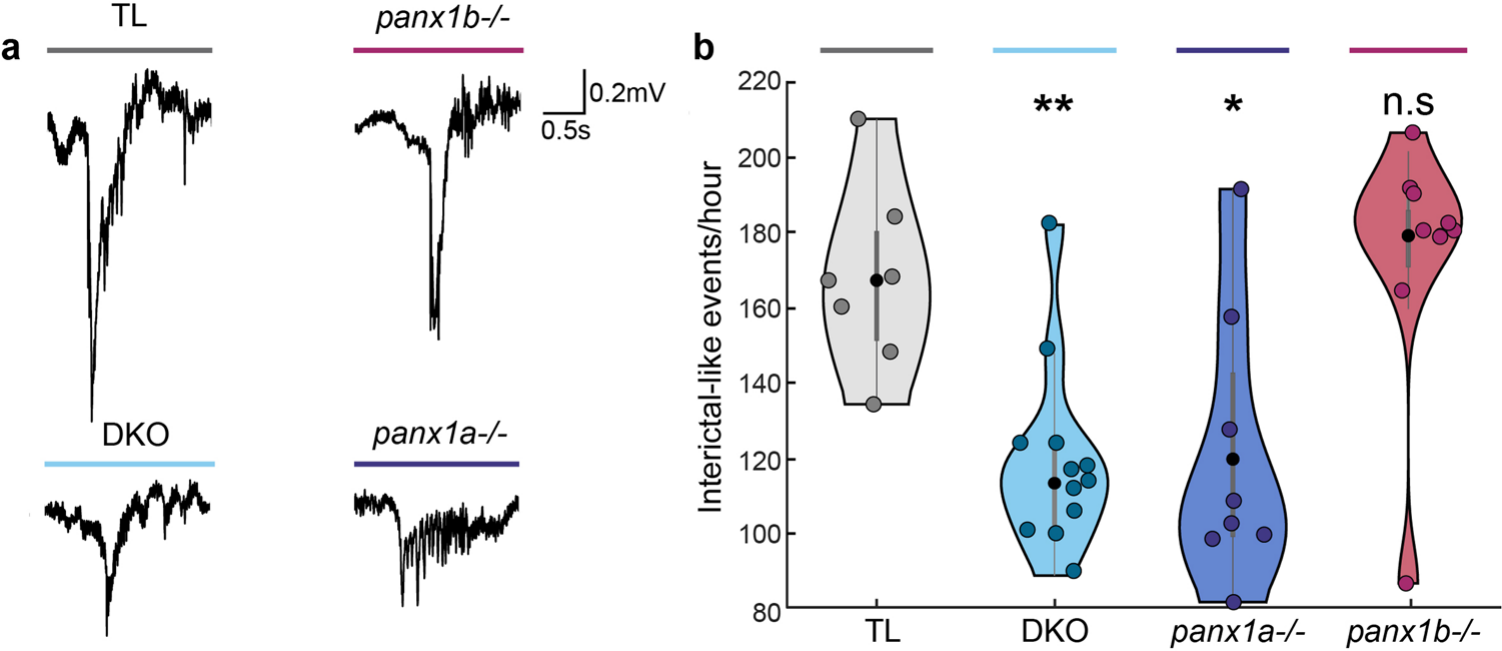
Interictal-like epileptiform discharges were decreased in the optic tectum of DKO and *panx1a*^*−/*−^ larvae. **a** Electrophysiological recordings of PTZ treatment showed interictal-like epileptiform discharges (IED) in all genotypes. Representative IEDs show most similarities between TL and *panx1b*^*−/−*^ events. **b** Quantification of IEDs for the first hour of recording for all genotypes revealed that DKO (*P* = 1.3 × 10^−3^, *n* = 12) and *panx1a*^−*/−*^ (*P* = 0.02, *n* = 8) had significantly fewer IEDs. No significant difference was found in the amount of IEDs for *panx1b*^−*/*−^ larvae compared to TL (*P* = 0.34, *panx1b*^*−/*−^ *n* = 9, TL *n* = 7). Data presented as an average number of events per hour ± s.e.m. N = number of larvae. Scale bar: 0.2 mV by 0.5 s. *P < 0.05, **P < 0.01, Mann–Whitney U test.

Interictal-like epileptiform discharges (IEDs) were investigated for genotype-dependent changes in occurrence and waveform. IEDs were defined as events that were shorter than 3 s and had amplitudes >1.5 times the baseline activity^42^. Figure 3a depicts typical IEDs, with TL and *panx1b*^−*/−*^ events appearing most similar. The quantification of IEDs during a 1 h PTZ application period showed that DKO (119 ± 7.2 events/hour; *P* = 0.0013) and *panx1a*^*−/*−^ (121.4 ± 12.9 events/hour; *P* = 0.0205) larvae exhibited IEDs. However, their IEDs occurred significantly less when compared to TL (167.3 ± 9.3 events/hour) and *panx1b*^−*/*−^ larvae (174.1 ± 11.5 events/hour; *P* = 0.3368); both TL and *panx1b*^*−/−*^ were similar (Fig. 3b).

### Targeting Panx1 improves PTZ-induced seizure locomotion and molecular responses

**Fig. 4:**
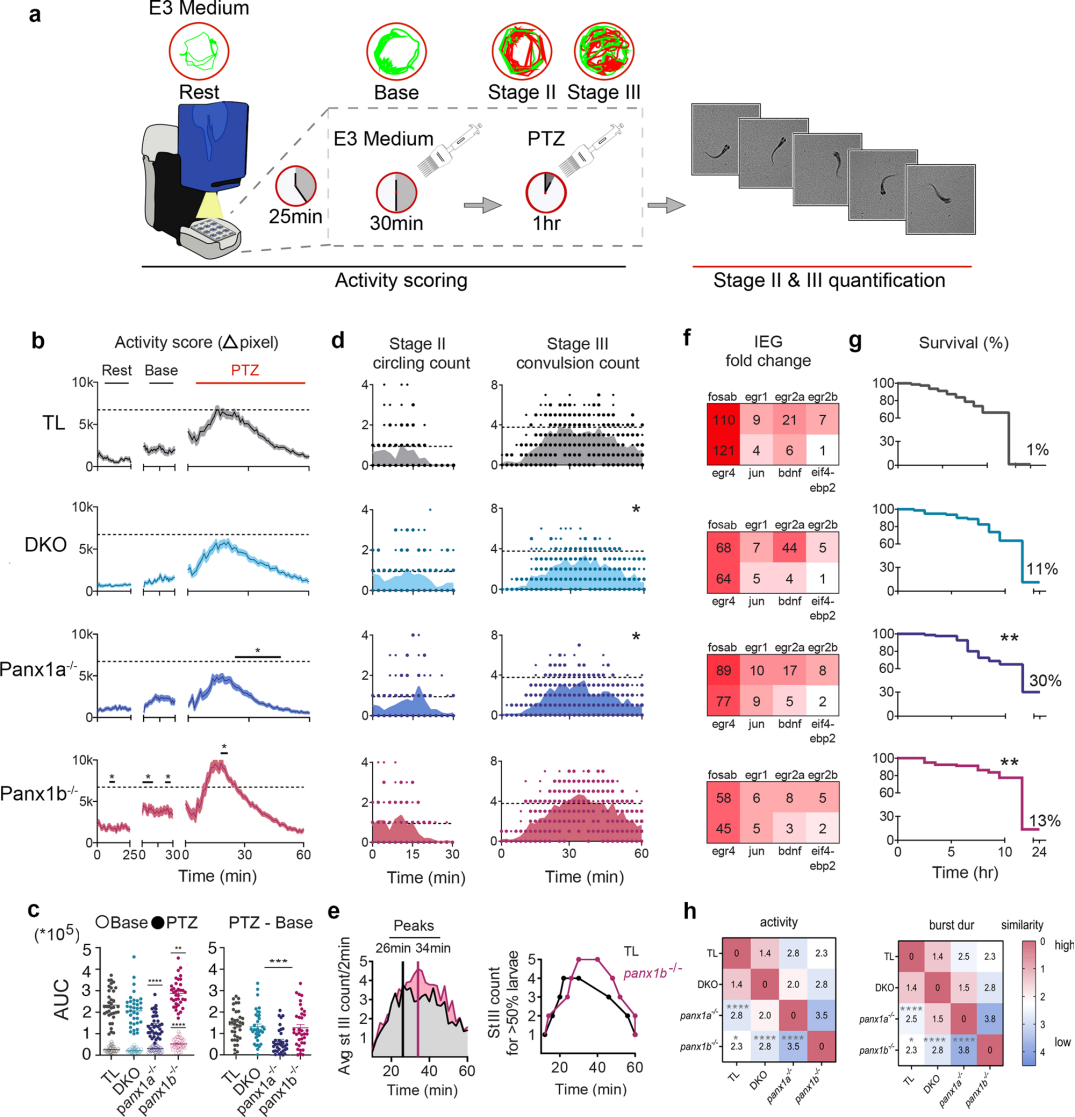
Behavioral and molecular outcomes of targeting *panx1* by gene-editing. **a** Methodological workflow of behavioral assays in TL and *panx1* knockout larvae. **b** Larvae’s (*n* = 36/genotype) baseline and 15 mM PTZ-induced activity (Δpixel ± s.e.m.) were scored. *Panx1b*^*−/−*^ baseline activity was higher than TL (*P* = < 1.0 × 10^−4^). PTZ induced hyperactivity peaked at 15–30 min for TL (gray), was reduced in DKO (light blue), significantly reduced in *panx1a*^−*/*−^ (dark blue, *P* = < 1.0 × 10^−4^) for the last 40 min, and significantly increased in *panx1b*^−*/*−^ larvae (magenta, *P* = 1.3 × 10^−3^) around 20 min compared to TL; Two-way repeated measures ANOVA and Bonferroni multiple comparisons. Dashed lines indicate TL’s max average activity score. **c** AUC derived from the activity plots in (**b**) significantly differed between TL and *panx1b*^*−/−*^ baselines (open points; *P* < = 1.0 × 10^4^). AUC for 1 h post-PTZ treatment was significantly reduced for *panx1a*^−*/*−^ (*P* = < 1.0 × 10^−4^) and increased for *panx1b*^−*/−*^ (filled points; *P* = 5.7 × 10^−3^). Change in PTZ treatment from average baseline activity was significantly reduced in *panx1a*^*−/−*^ compared to TL (*P* = 2.0 × 10^−4^; Tukey’s multiple comparisons test). **d** Stage II count (rapid circling) did not differ among groups (*n* = 18; count/2 min). Stage III (convulsion) was significantly reduced in DKO (*P* = 1.5 × 10^−2^) and *panx1a*^*−/*−^ (*P* = 0.02; Two-way repeated measures ANOVA) compared to TL. Dashed lines represent max average stage II and stage III counts for TL. **e** Time course of average stage III counts differed between the two groups, peaking at 26 min post-PTZ treatment for TL and 34 min for *panx1b*^*−/*−^. Time course where >50% of the larvae reached 1–5 stage III counts revealed a delayed peak onset for *panx1b*^−*/−*^. **f** IEG upregulation after 1 h PTZ treatment, represented as fold-change for PTZ treated larvae against non-treated controls. *Panx1* knockout larvae had reduced upregulation of the IEGs compared to TL, with DKO and *panx1b*^−*/*−^ showing the greatest reduction. (*N* = 3 experimental replicates). **g** Survival rate of larvae (*n* = 80) post-PTZ treatment was significantly higher for *panx1a*^−*/*−^ followed by *panx1b*^*−/−*^, and the survival rate of DKOs did not differ from TL. Survival at 24 h: TL: 1.25%, DKO: 11%; *P* = 0.38, *panx1a*^*−/*−^: 30%; *P* = 8.8  × 10^−3^, *panx1b*^*−/*−^: 13%; *P* = 8.1 × 10^−3^; Mantel–Cox test. **h** SOM revealed similarities and differences in PTZ-induced activity and bursting behaviors of TL and *panx1* knockouts (*n* = 36). TL and DKO were most similar, *panx1a*^−*/*−^ differed the most from TL (*P* < 0.0001; Fisher’s exact test), and *panx1a*^−*/*−^ and *panx1b*^−*/*−^ were most distinct (*P* < 0.0001). Values and color represent the degree of similarity. N = number of larvae. *P < 0.05, **P < 0.01, ***P < 0.001, ****P < 0.0001.

Locomotion tracking was used to quantify genotype-specific seizure-related behaviors in response to 15 mM PTZ treatment (Fig. 4a). Activity scores (Δpixel) for locomotor activity were plotted for: rest, baseline, and post-PTZ treatment (*n* = 36 per genotype; Fig. 4b). At the start of the baseline recording, the addition of fresh E3 medium caused a transient minor activity increase for all larvae, which stabilized within 30 min. PTZ treatment increased locomotor activity significantly in all genotypes. TL larvae showed a continuous activity increase in the first 10 min, a peak around 15–30 min, and a gradual decline within the hour. TL and DKO activity curves were statistically similar. The *panx1a*^*−/−*^ activity curve was significantly reduced compared to TL controls after the transient peak observed 20 min post-PTZ treatment (*P* < 0.0001). The *panx1b*^*−/−*^ larvae exhibited a sharp spike in activity between 10 to 20 min, with a steeper decline than TL (*P* = 0.0013).

PTZ-induced hyperactivity was quantified from the area under the curve (AUC) shown in Fig. 4b (Fig. 4c). The baseline AUC was significantly larger in *panx1b*^*−/*−^ larvae relative to TL in the last 15 min of stabilized baseline activity (*P* < 0.0001). The AUC post-PTZ treatment, taken over 60 min, was greater in *panx1b*^*−/−*^ (*P* = 0.0057) and reduced in *panx1a*^−*/−*^ (*P* < 0.0001) larvae relative to TL. When the mean baseline activity was extracted from the post-PTZ treatment AUC, only *panx1a*^−*/*−^ larvae activity was significantly reduced compared to TLs (*P* = 0.0002).

Next, the consecutive stages of seizure-like behavior were analyzed (Supplementary Movies 1, 2). Stage II, a rapid ‘whirlpool-like’ circular movement, and stage III, uncontrollable clonus-like twitching of the body followed by a prolonged loss of posture, were scored manually via video recordings of the fish. Stage II and III events were quantified in 2 min intervals for 1 h of PTZ treatment (*n* = 18 per genotype; Fig. 4d). Stage II onset occurred within a few minutes of treatment and lasted for 30 min. TL and *panx1b*^−*/*−^ larvae entered Stage II first and peaked at ≈10 min post-treatment. Stage II activities for DKO and *panx1a*^*−/*−^ larvae occurred throughout the first 30 min. The onset and total count of stage II locomotion did not significantly differ between genotypes (DKO, *P* = 0.0982; *panx1a*^*−/*−^, *P* = 0.328; *panx1b*^*−/*−^, *P* = 0.080). Stage III convulsive behavior started after 10 min of PTZ treatment for all genotypes. Peak stage III activity was observed within 20–40 min, followed by a gradual decline. Stage III events in DKO (*P* = 0.0149) and *panx1a*^−*/−*^ (*P* = 0.0234) larvae were significantly reduced compared to TL controls. The peak activity latency, calculated at the time point with the highest average of stage III activity, was delayed for *panx1b*^−*/*−^ larvae (34 min) compared to TLs (26 min; Fig. 4e). Furthermore, time points at which 50% or more larvae entered stage III, indicated a delayed onset and termination of stage III in *panx1b*^*−/−*^ (Fig. 4e). We speculate that the latency differences in the onset of electrographic seizures and behavioral outcomes are explained best by how loss-of Panx1 functions influence the molecular basis of synaptic excitability and how these alterations affect pathways leading to a common behavioral output. These differences in latency are more prominent when measuring electrographic activity than behavioral outputs^43^ and are consistent with previous reports regarding the seizures in the Panx1 knockout mice^8^.

Differential expression of Immediate Early Genes (IEGs) was tested after the behavioral assays’ experimental endpoints were reached. The IEGs were selected based on previously reported robust activation in a zebrafish PTZ model^36^, human epileptic neocortex^44^, and rodent models^45,46^. Figure 4f shows a 100-fold increase in the expression of *fosab* and *egr4* (in red) in PTZ treated TLs, which was twice as high compared to the fold change observed in DKO and *panx1b*^*−/−*^ larvae. DKO and *panx1b*^*−/−*^ larvae showed a modest upregulation for *fosab, egr1, egr2b, egr4, bdnf*. In *panx1a*^−*/*−^ larvae, upregulation of *fosab, egr2a, egr4, bdnf* was moderate, but *egr1, egr2b*, and *jun* showed a strong differential upregulation (Supplementary Table 1).

Kaplan–Meier plots show the larvae survival, which was determined by monitoring blood circulation and heartbeat post-PTZ treatment for 10 consecutive hours and at 24 h (*n* = 80 per genotype; Fig. 4g). Although the *panx1a*^−/−^ group declined between 5 and 10 h, they had the highest survival rate of 30% at 24 h (*P* = 0.0088). The *panx1b*^*−/*−^ group’s survival rate was stable for the first 10 h and declined to 13% after 24 h (*P* = 0.0081). The low DKO and TL survival rate at 24 h were statistically similar.

Self-Organizing Maps (SOM) for unsupervised clustering of behavioral data for PTZ treated TL and *panx1* knockouts (*n* = 36 per group; Fig. 4h) showed that activity scores (∆pixel), bursting behavior count, and duration (sec) displayed the most robust differences between the genotypes among the eight behavioral outputs recorded; TL and DKO groups exhibited the highest behavioral similarity, while *panx1a*^*−/−*^ was most different to TL (*P* < 0.0001). *Panx1a*^*−/−*^ and *panx1b*^*−/−*^ larvae showed the most distinct activity and burst duration (*P* < 0.0001). Freezing and other locomotor behaviors varied less among genotypes (Supplementary Fig. 4). The SOM approach signified the heightened activity, specifically bursting behavior, in the PTZ-induced behavioral phenotypes.

### Acute pharmacological blocking of Panx1 suppresses seizure-like activities

**Fig. 5:**
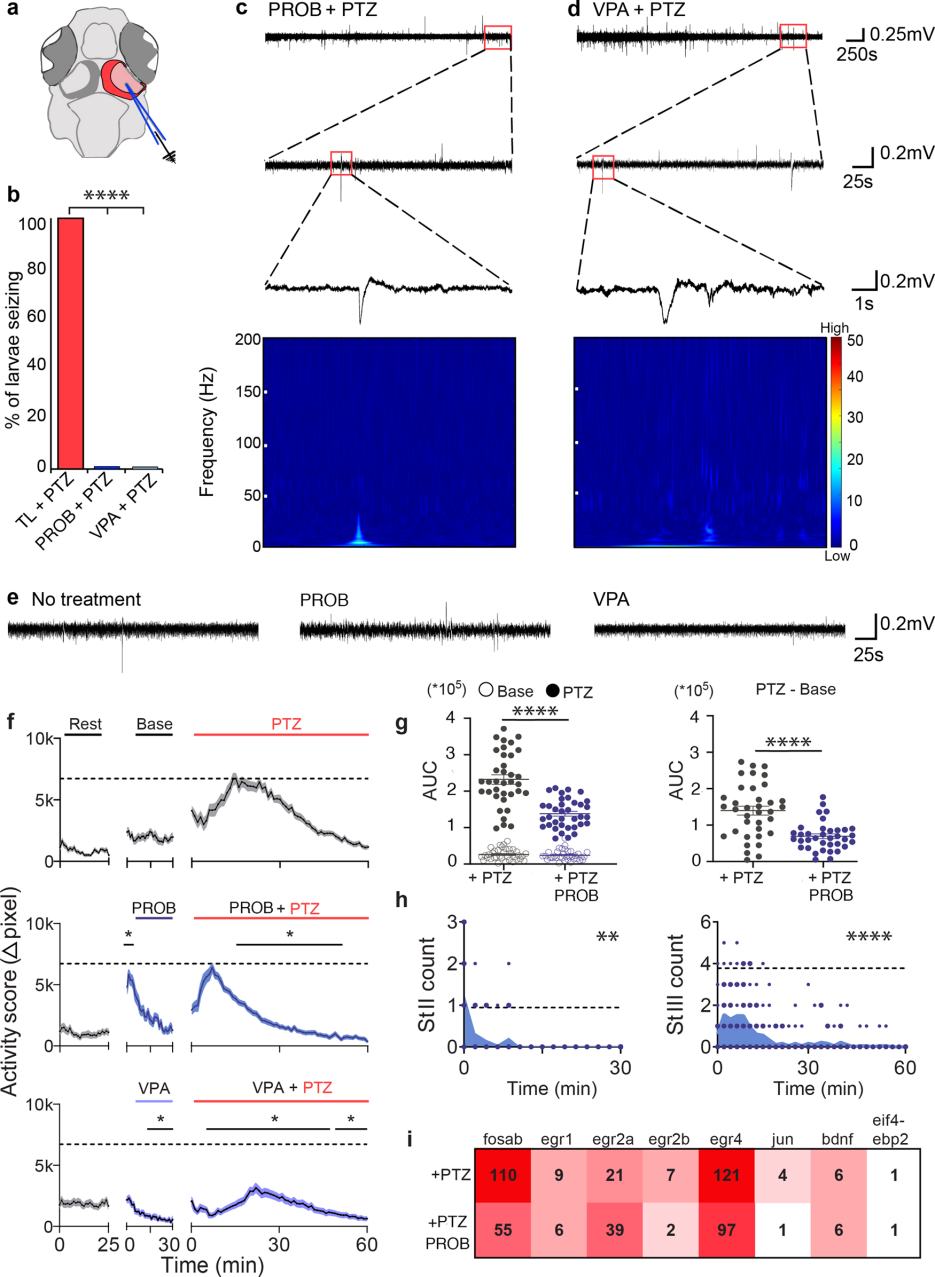
Acute blocking of Panx1 with PROB effectively prevents seizure-like activity. **a** Schematic of the region LFPs were recorded from. **b** Larvae treated with 75 µM PROB (*n* = 7; *P* = 6.1 × 10^−5^) or 5 mM VPA (*n* = 7; *P* = 6.1 × 10^−6^) have no PTZ inducible SLEs compared to 100% of TL larvae (*n* = 7; Barnard test). Representative traces of larvae treated with (**c**) PROB and (**d**) VPA treated with PTZ for 60 min. Expanded views into the last 5 min of traces showed lack of SLEs. Spectrograms corresponding to small spikes in traces above demonstrate no increase in high-frequency power associated with these events. Scale bar: A value of 0 (blue) is low and a value of 50 (red) is high. **e** LFPs of the last 5 min of 60 min traces revealed no drug induced changes to baseline activity when using PROB or VPA. **f** Baseline activity (∆pixel ± s.e.m.; *n* = 36; Two-way repeated measures ANOVA and Bonferroni multiple comparisons) increased with PROB treatment for the first 5 min (*P* = < 9.0 × 10^−4^), PTZ-induced hyperactivity subsided significantly within 15 min (*P* = < 1.0 × 10^−4^), sooner than the PTZ only group. VPA treatment (*n* = 60) decreased baseline and PTZ-induced activity (*P* = < 1.0 × 10^−4^), with a hyperactivity curve like the PTZ only group. Dashed lines indicate max average activity for PTZ only group. **g** AUCs for baseline activity and PROB treatment did not differ (open points; *P* = 0.5). PROB treatment significantly reduced the effect of PTZ without (filled points; *P* = < 1.0 × 10^−4^) and with (*P* = < 1.0 × 10^−4^; Unpaired *t*-test) extracted baseline activity. **h** Stage II and III counts (*n* = 18; count/2 min) were significantly reduced with PROB treatment (stage II: *P* = 5.7 × 10^−3^; stage III: *P* ≤ 1.0 × 10^−4^; Two-way repeated measures ANOVA), majority occurring in the first 10 min of treatment. Dashed lines represent max average Stage II and Stage III counts for PTZ-only group. **i** IEG upregulation was reduced in PROB treated TL larvae except for *egr2a*. N = number of larvae. Scale bars: Top = 0.25 mV by 250 s, middle = 0.2 mV by 25 s, bottom = 0.2 mV by 1 s, **e** = 0.2 mV by 25 s. *P < 0.05, **P < 0.01, ***P < 0.001, ****P < 0.0001.

Acute changes to electrophysiological discharges and behavioral and molecular outcomes in the presence of PTZ were determined after treatment with PROB, a well-established Panx1 channel blocker^47^. Valproic acid (VPA), a common anticonvulsant, served as a control for suppressing SLEs^48^. All TL larvae were treated with PROB (75 µM) or VPA (5 mM) for 10 min prior to PTZ application (Fig. 5a). For the duration of PTZ treatment, SLEs in the OT were suppressed in all PROB (*n* = 7) and VPA (*n* = 7) treated larvae (Fig. 5b) as shown in selected recordings (Fig. 5c, d). Traces from time points near the experimental endpoint were depicted at a higher resolution to show the more considerable deflections. The spectral analysis of the deflections revealed that no increase in high-frequency power was associated with this type of activity. LFPs recorded from larvae exposed to PROB or VPA without PTZ confirmed that PROB was not suppressing SLEs due to toxicity. The electrographic activity was comparable amongst these groups (Fig. 5e, Supplementary Fig. 5).

Locomotor activity was monitored for TL larvae treated with either 75 µM PROB (*n* = 36) or 5 mM VPA (*n* = 60) and compared to untreated controls (Fig. 5f). PROB application alone induced a brief, but sharp, increase in larvae activity that returned to baseline within 30 min (Fig. 5f middle; *P* = 0.0009). VPA treatment caused reduced activity, which decreased below baseline after 10 min (Fig. 5f bottom; *P* < 0.0001). After a 30 min baseline recording, all larvae were treated with PTZ for 60 min. PROB treated larvae had an early onset of spiking activity within the first 10 min after PTZ treatment, ahead of larvae treated with PTZ only. PROB treated PTZ-induced activity subsided within 30 min and returned to baseline, significantly faster than PTZ only treated larvae (*P* < 0.0001). VPA treatment significantly reduced PTZ-induced activity, with a timeline and profile similar to the PTZ-only group (*P* < 0.0001). AUC analysis on activity plots in Fig. 5f showed that baseline AUC did not significantly differ between no treatment and PROB treated larvae in the last 15 min of stabilized activity (Fig. 5g; *P* = 0.502). Post-PTZ treatment AUC, with or without extracted baseline activity, was larger in the PTZ-only treated group than in the PROB-PTZ treated group (*P* < 0.0001). The analysis of seizure-related Stages II and III of PROB treated larvae (*n* = 18) every 2 min for a duration of 60 min (Fig. 5h) showed that Stage II activity was significantly reduced compared to PTZ-only treated larvae, with most of the activity occurring in the first 10 min (*P* = 0.0057). Stage III activity of PROB-treated larvae displayed an immediate onset compared to the PTZ-only group and was significantly reduced after 20 min of PTZ treatment (*P* < 0.0001). The differential expression of IEGs after PROB treatment was consistent with the changes observed in gene-edited larvae; IEG expression was reduced after PROB treatment compared to the PTZ only treated group (Fig. 5i). PROB treated TL larvae exhibited IEG expression like DKOs, where all IEGs exhibited less upregulation, except for *egr2a*. *Fosab’s* upregulation was reduced by twofold, and *jun* had no significant upregulation with PROB treatment (Supplementary Table 2).

### Seizure phenotypes are linked to transcriptome changes

**Fig. 6:**
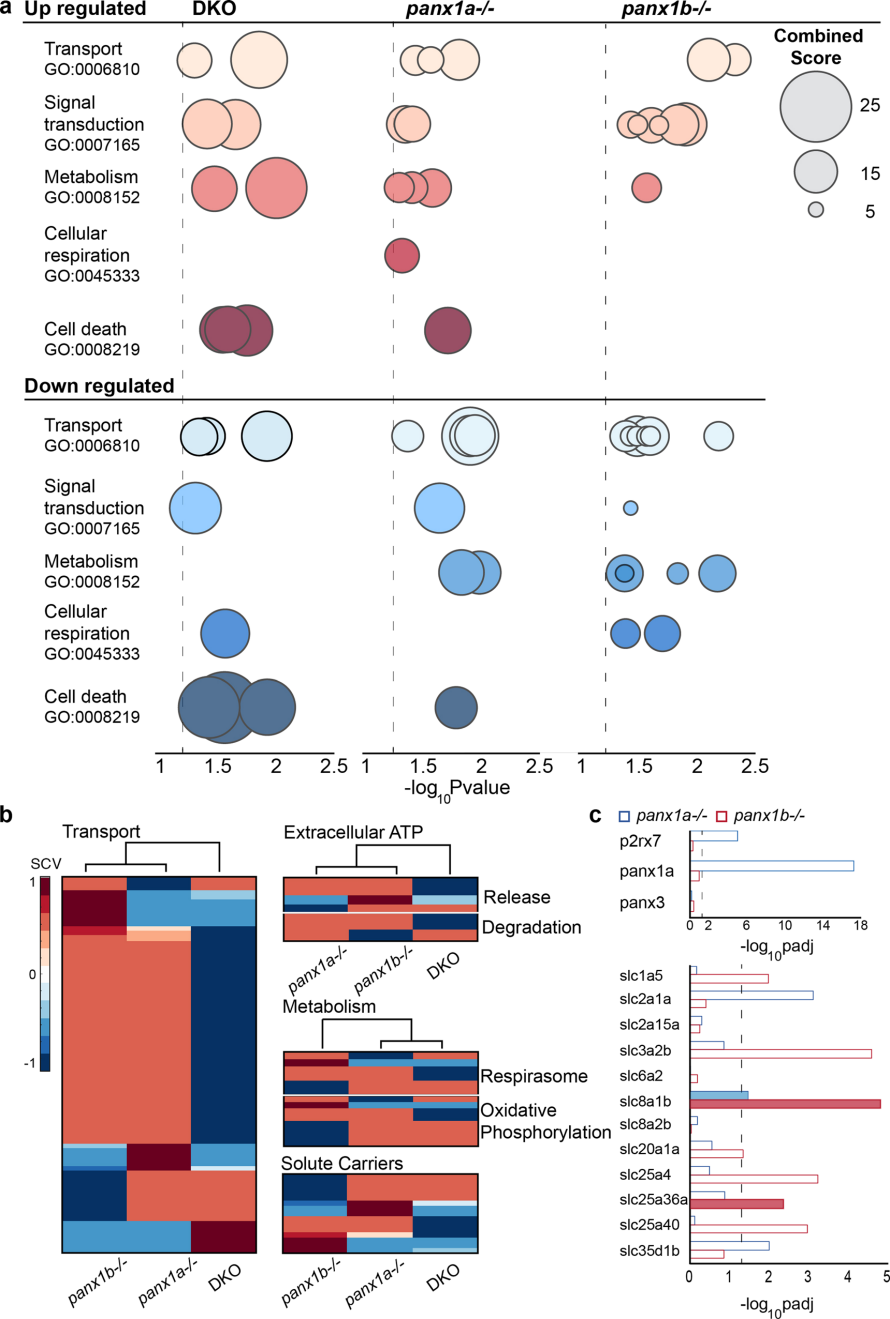
Loss of *Panx1a* affects biological processes related to metabolism and transport. **a** Gene ontology enrichment of biological processes for differentially regulated genes from RNA-seq data of DKO, *panx1a*^−*/−*^, and *panx1b*^−*/−*^ found using the FishEnrichR database. Biological processes were grouped into five categories; transport (GO:0006810), signal transduction (GO:0007165), metabolism (GO:0008152), cellular respiration (GO:0045333), and cell death (GO:0008219). Data is presented as -log *P* value based on Fisher’s exact test. The dot size represents the combined score for genes associated with that pathway. Dotted gray lines indicate *P* = 0.05. **b** Candidate genes were selected, and RNA-seq data was mined for differential regulation in panx1 fish lines. Clustergrams compare *panx1* knockout larvae for correlation amongst chosen genes for (1) transport, (2) extracellular ATP (release & degradation), (3) metabolism (respirasome & oxidative phosphorylation) and (4) solute carriers. The scale bar shows standardized correlation values (SCV) calculated by the Matlab Clustergram function. Range: red is positive, and blue is negative. Hierarchical cluster branches are shown above each clustergram and labeled below. **c** Expression (logpadj) of genes part of the ATP release (top) and solute carrier (bottom) clustergrams for *panx1a*^−*/*−^ (blue) and *panx1b*^*−/*−^ (top). Filled bars indicate upregulated genes; empty bars are downregulated. *P* = 0.05 indicated with dotted line.

RNA-seq data of untreated 6dpf larvae^32,39^ showed differences in the number and regulation of genes when the FishEnrichR database^49,50^ was data-mined for biological processes enriched in the nervous system (Supplementary Fig. 6). The GO biological process categories selected for further analysis represented broad themes based on known (signal transduction, cell death, transport) or anticipated (metabolism, cellular respiration) roles of *Panx1* channels (Fig. 6a, Supplementary Data 1).

All genotypes shared enrichment of the categories, *transport* (GO:0006810) and *signal transduction* (GO:0007165). The lack of enrichment of *cellular respiration* (GO:0045333) in *panx1b*^−*/−*^ larvae suggested that mitochondrial energy metabolism processes and response to reactive oxygen species were less controlled after losing *panx1b*. Further, *panx1b*^*−/*−^ larvae stood out for lacking enrichment of *cell death-associated processes* (GO:0008219). The opposite enrichment of the same categories in *panx1a*^−*/*−^ larvae repeated the pattern of electrographic and behavioral activities. DKOs displayed a mixed phenotype, lacking a downregulated metabolism (GO:0008152).

Cluster analysis linked coordinated expression changes with *Panx1* genotypes (Fig. 6b). Genes were manually curated based on the enriched biological processes (Supplementary Data 2). Genotype-specific transport and metabolic properties corroborated the pattern of shared and distinct roles of zebrafish *panx1* ohnologues. *Panx1a*^*−/*−^ and *panx1b*^*−/−*^ larvae had the opposite coordination of gene expression changes in subcategories of metabolism, mitochondrial ATP production, and oxidative phosphorylation processes. However, the coordinated expression was strengthened for genes encoding proteins that release or degrade ATP in *panx1a*^*−/*−^ and *panx1b*^*−/−*^ larvae. Interestingly, transport genes showed similar enhanced coordination in both genotypes, consistent with the roles of both channel proteins in cell signaling. However, coordinated expression of the solute carrier (SLC) group of membrane transport proteins involved in signal transduction pathways showed more significant variation between genotypes consistent with the GO ontology enrichment analysis; overall, DKOs had a less coordinated expression in these categories.

A direct comparison of *panx1a*^−*/*−^ and *panx1b*^*−/*−^ larvae corroborated the significant downregulation of *panx1a* and *p2rx7* expression as the signature difference between the two genotypes (Fig. 6c). Interestingly, the *slc2a1a* was downregulated in *panx1a*^*−/*−^ larvae. In Mammalia, this glucose transporter is in the blood-brain barrier. Pathogenic SLC2A1 variants are associated with epilepsy^51^. In *panx1b*^*−*^^/*−*^ larvae, SLC proteins with molecular functions in Na^+^/Ca^2+^ transmembrane transport (*slc8a1b*) or as pyrimidine nucleotide transmembrane transporters (*slc25a36a*) were upregulated, while regulation of sodium-dependent amino acid transporters (*slc1a5*), mitochondrial ADP/ATP antiporters (*slc25a4*), or transport across the inner membrane of mitochondria (*slc25a40*), was reduced. Although the exact function of the upregulated *slc3a2b* in the zebrafish is unknown, in humans, heterodimers of SLC3A2 with the large neutral amino acid transporter (LAT1) function as sodium-independent, high-affinity transporters that mediate uptake of large neutral amino acids such as phenylalanine, tyrosine, L-DOPA, leucine, histidine, methionine, and tryptophan. Although the analysis presented only a snapshot of relevant molecular signatures, we concluded that the differences in prominent biological processes primed each genotype’s response to PTZ differently.

### Evidence for an ATP-dependent mechanism contributing to seizure-like activity

**Fig. 7:**
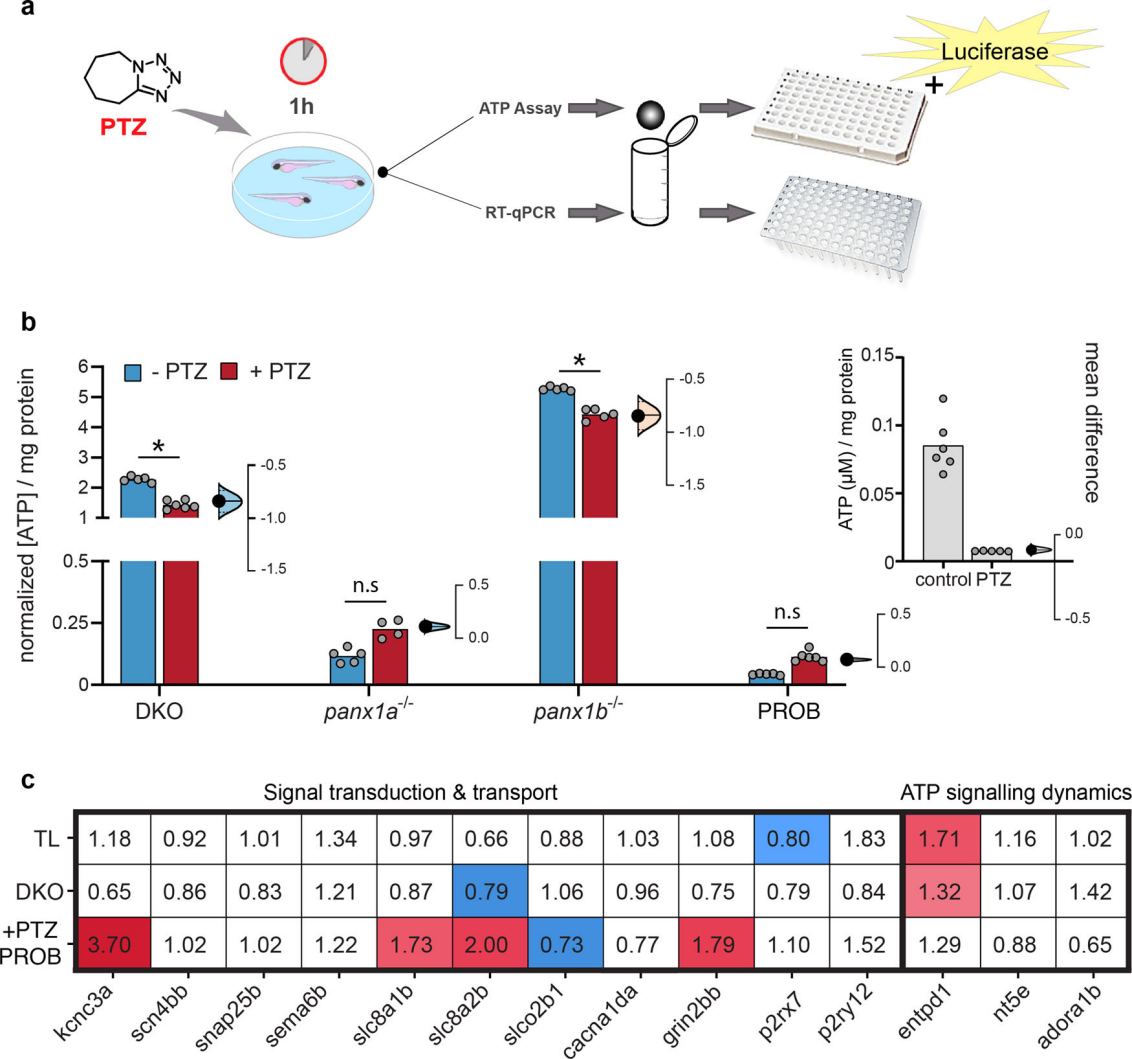
Extracellular ATP is associated with a propensity for seizure-like activity. **a** Workflow diagram outlining the 1 h PTZ incubation for treated larvae. Larvae were collected for either RT-qPCR (*n* = 30 larvae/sample, *N* = 3 experimental replicates) or ATP assays (*n* = 50 larvae/sample, *N* = 5 experimental replicates), followed by homogenization prior to samples being plated in 96well format for measurement. Note that for ATP detection, luciferase was used. **b** Estimation plots of extracellular ATP concentrations (µM) concerning larval protein content (mg/ml) and normalized to the respective TL control. Extracellular ATP for TL controls is shown on the right (**b**), showing a significant decrease in ATP with PTZ treatment (*P* = < 1.0 × 10^−4^). Baseline ATP values are depicted by the blue bars, PTZ treatment is in red, and individual data points are displayed in gray to show data distribution. Difference of means is shown to the right of each group, with a 95% confidence interval to show effect size and significance. DKO (Δ mean = −0.84) and *panx1b*^−*/−*^ (Δ mean = −0.84) show a significant decrease in ATP with PTZ treatment. *Panx1a*^−*/*−^ (Δ mean = 0.1) and TL larvae with PROB (Δ mean = 0.07) show very low ATP concentrations; however, they do show a slight increase in ATP with PTZ treatment which is not significant. *Panx1b*^−*/*−^ has the highest presence of ATP. (DKO: *P* = < 1.0 × 10^−4^; *panx1a*^*−/*−^: *P* = 0.98; *panx1b*^*−/*−^: *P* = < 1.0 × 10^−4^; PROB: *P* = 1.0; Tukey’s multiple comparisons test). **c** RT-qPCR of selected genes grouped into signal transduction and transport, or ATP signaling dynamic categories, showing significant up (red) or down (blue) regulation, with respect to non-treated controls, in response to PTZ treatment for TL, DKO and TLs treated with PROB. (*N* = 3 experimental replicates). *P < 0.0001.

Changes to ATP release and differential expression of biomarkers in response to PTZ conditions were quantified (Fig. 7a). Extracellular ATP concentrations were normalized to TL controls (gray bars) with and without PTZ treatment (baseline)^52,53^. Baseline extracellular ATP (blue bars) was significantly reduced for *panx1a*^*−/*−^ and PROB-treated TL larvae (Fig. 7b). PTZ treatment (red bars) did not change extracellular ATP for *panx1a*^−*/*−^ and PROB-treated TL larvae, consistent with reduced seizure-like activities. In DKO and *panx1b*^*−/*−^ larvae, Extracellular ATP was significantly elevated overall and was substantially altered by PTZ treatment. Our results suggested that the propensity to develop SLEs was correlated with the availability of extracellular ATP.

Reduced SLEs in gene-edited DKOs and pharmacological blocked TL was not simply correlated with low extracellular ATP. This unexpected difference was corroborated by the differential expression of selected biomarkers for signal transduction and transport (Fig. 7c, Supplementary Table 3). PROB treated TL larvae showed an upregulation of voltage-sensitive potassium channels required for rapid repolarization of fast-firing brain neurons (*kcnc3a*), Ca^2+^/Na^+^ antiporters (*slc8a1b* & *slc8a2b*), and NMDA receptors involved in excitatory postsynaptic potentials (*grin2bb*). DKOs had a significant downregulation of *slc8a2b*, an important Ca^2+^/Na^+^ antiporter found in axons and at the post synapse that plays a large role in Ca^2+^ and Na^+^ signaling and cell communication. However, PTZ treatment did not impact the expression of genes associated with extracellular ATP processing, signal transduction, or transport. The regulation of *entpd1*, which encodes for the rate-limiting ATP/ADP-hydrolyzing ectoenzyme CD39, appeared notable, but the expression of *p2rx7, p2ry12, nt5e*, or *adora1b* were similar in DKOs and PROB-treated TL. The results advocate for a role of *panx1a* in ATP metabolism and signaling, causing genotype-specific outcomes of seizure dynamics, and allude to alternative mechanisms for acute versus chronic *panx1* targeting.

#### *P2rx7* affects seizure-like activities but does not supersede *panx1a*

**Fig. 8:**
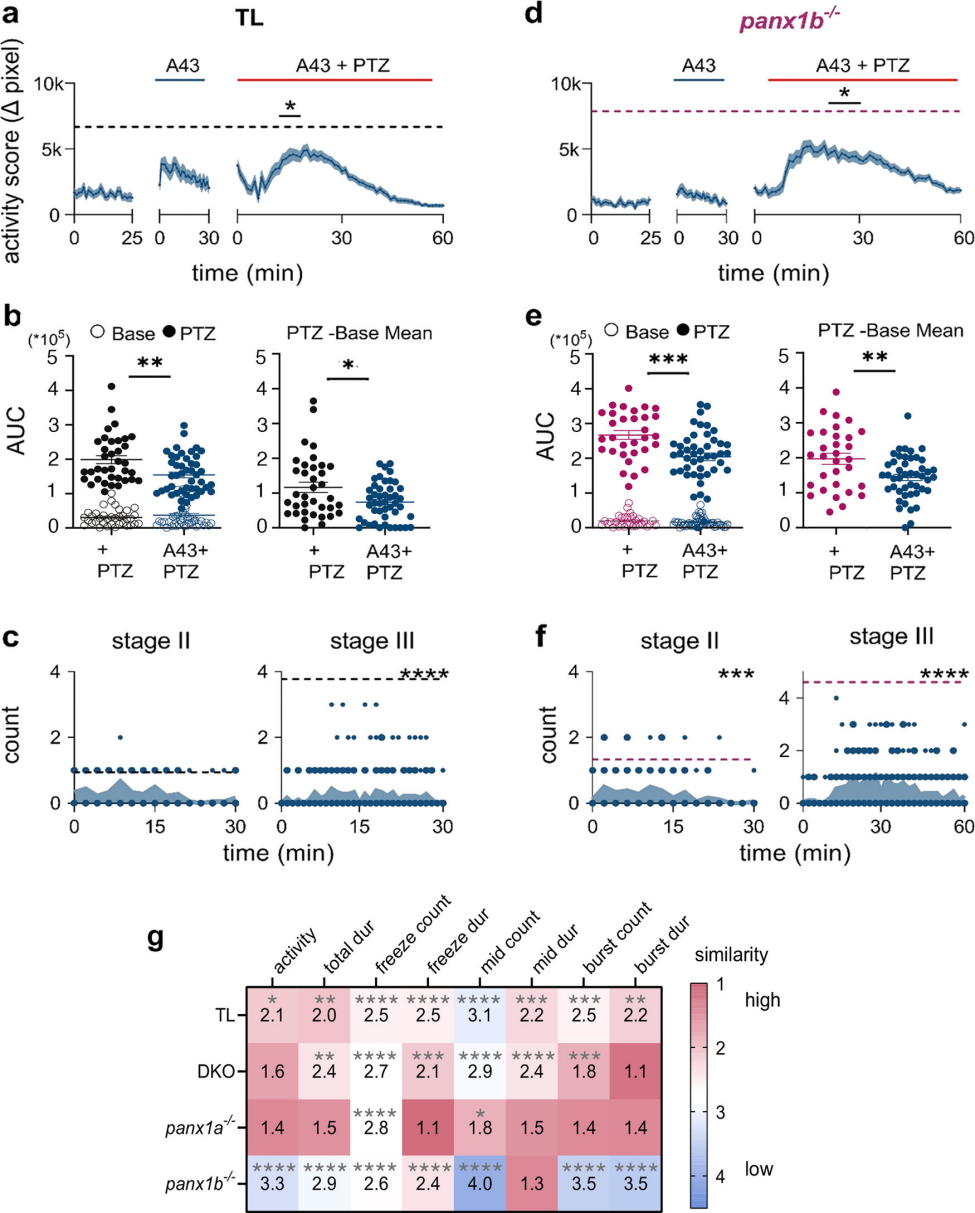
Blocking of P2rx7 with A-438079 improves seizure-like behavior. **a** 100 µM A-438079 (A43) treatment decreased hyperactivity in TL in the first 10 min of PTZ treatment (∆pixel ± s.e.m.; *n* = 46; *P* = 2.1 × 10^−3^). The dashed gray line indicates TL’s max average activity score treated with only PTZ (*n* = 36). **b** AUCs for TL’s baseline activity and A43 treatment did not differ (open points; *P* = 0.9). A43 treatment significantly reduced the effect of PTZ without (filled points; *P* = 2.4 × 10^−3^) and with (*P* = 4.5 × 10^−2^) extracted baseline activity. **c** Stage III count (count/2 min) was significantly reduced with A43 treatment in TL (*n* = 18; *P* = < 1.0 × 10^−4^). Dashed lines represent max average Stage II and Stage III counts for TL’s PTZ-only group. **d** A43 treatment decreased PTZ-induced hyperactivity in *panx1b*^*−/*−^ (*n* = 46; *P* = 4.0 × 10^−4^). The dashed magenta line indicates *panx1b*^*−/*−^’s max average activity in the PTZ only group (*n* = 31). **e** A43 treatment significantly reduced the effect of PTZ in *panx1b*^*−/−*^ without (filled points; *P* = 5.0 × 10^−4^) and with (*P* = 8.7 × 10^−3^) extracted baseline activity. **f** Stage II and III counts were significantly reduced with A43 treatment in *panx1b*^−*/*−^ (*n* = 18; stage II: *P* = 1.0 × 10^−4^; stage III: *P* ≤ 1.0 × 10^−4^). Dashed lines represent max average Stage II and Stage III counts for *panx1b*^*−/*−^’s PTZ-only group. **g** SOM revealed changes to the PTZ phenotype in TL with A43 treatment (*n* = 46) for freezing and normal locomotor behaviors (*P* < 0.0001). A43 treatment resembled *panx1a*^−*/−*^‘s PTZ phenotype the most, and *panx1b*^−*/−*^‘s the least, as seen in normal (mid count *P* < 0.0001) and bursting behaviors (*P* < 0.0001). Smaller value and warmer color represent higher similarity. Statistical tests used: Two-way repeated measures ANOVA and Bonferroni multiple comparisons for(**a**, **c**, **d** & **f**); Mann–Whitney U test for (**b** and **e**). Fisher’s exact test for (**g**). N = number of larvae. *P < 0.05, **P < 0.01, ***P < 0.001, ****P < 0.0001.

The impact of P2rx7 on seizure activity in the presence of both *panx1* genes was tested in TL larvae. A-438079 hydrochloride hydrate (100 µM A43; *n* = 46) was applied 30 min before PTZ application without altering the larvae’s activity. PTZ-induced hyperactivity was reduced in A43 treated TL larvae compared to the activity induced in PTZ treated TLs (gray dotted line; *n* = 36; *P* = 0.0021) (Fig. 8a). The AUC analysis corroborated that treatment with both A43 and PTZ significantly reduced the AUC when compared to PTZ treated larvae, with (*P* = 0.0448) and without (*P* = 0.0024) extracted baseline activity (Fig. 8b). A43 treatment also significantly reduced Stage III counts (*n* = 18; Fig. 8c; *P* < 0.0001) without influencing stages II and III’s onset and peak activity in the test period.

To identify a potential role of *panx1a* in seizure-like activity, *panx1b*^−*/*−^ larvae that express *panx1a* and *p2rx7* mRNA were treated with A43 (*n* = 46). The A43 treatment alone did not affect the baseline locomotor activity. However, PTZ-induced activity was significantly reduced after 20 min compared to larvae treated with PTZ only (magenta dotted line; *n* = 31; *P* = 0.0004) (Fig. 8d). The AUC analysis confirmed that A43 treated *panx1b*^*−/*−^ larvae had a reduced AUC than the PTZ treated group (Fig. 8e; *P* = 0.0005). A43 treatment also reduced Stage II (*P* = 0.0001) and III (*P* < 0.0001) event counts significantly (*n* = 18; Fig. 8f). Our results demonstrated that the pharmacological targeting of P2rx7 in both TL and *panx1b*^−*/−*^ larvae improved outcomes of PTZ induced seizure-like swimming behavior. Furthermore, Panx1a appears to play a predominant role in the seizure-like activity.

Next, the treatment of TL with both A43 and PTZ (*n* = 46) was examined using SOM (Fig. 8h). The A43 treatment altered the PTZ phenotype, specifically in freezing and normal locomotion behaviors (freeze count, *P* < 0.0001; mid count, *P* < 0.0001; for *panx1a*^*−/*−^ *P* = 0.016). The phenotype induced was most similar to *panx1a*^−*/−*^*‘s* and most distinct from *panx1b*^*−/*−^*‘*s (*P* < 0.0001 except mid-duration *P* = 0.055). Robust differences were seen in general activity, bursting, and normal locomotor behaviors.

#### Molecular modeling of fish and human pannexins

High-resolution cryo-EM structural studies of human and frog Panx1 have provided unanticipated outcomes^54–57^. Panx1 forms heptameric channels in contrast to the hexameric organization of connexons and the octameric organization of innexins. A carboxy-terminal region with known regulatory activity was not presented, and thus, its structure-function relationship could not be determined. Furthermore, many mechanistic questions remain concerning gating and selection of ions despite having a high-resolution view of the channel.

**Fig. 9:**
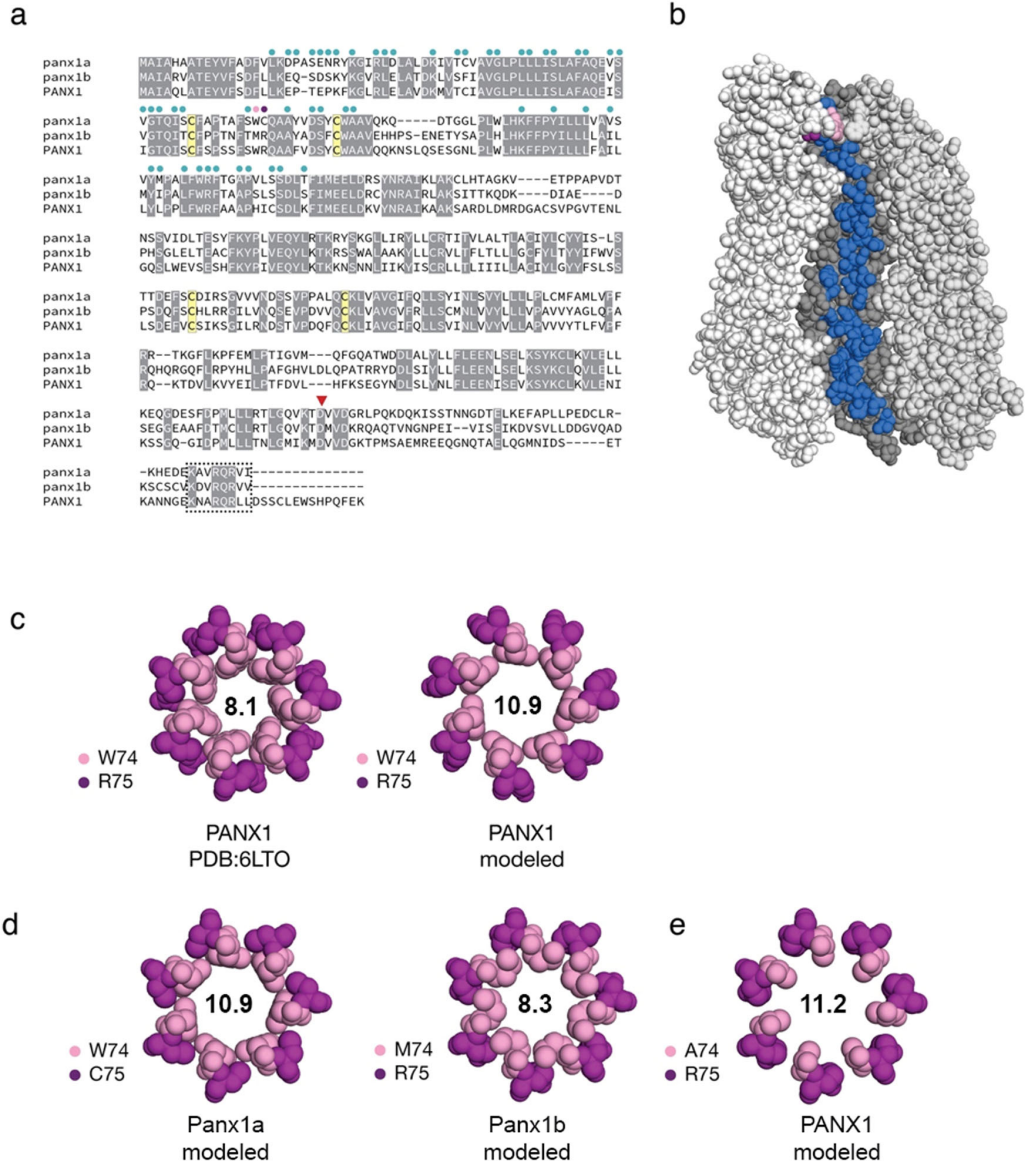
Sequence and structural attributes of the zebrafish pannexins. **a** Protein sequence alignment of full-length human pannexin-1 (PANX1; amino acids (aa): 1-426) and zebrafish Panx1a (aa 1-416), and Panx1b (aa 1-422). Circles above the sequence alignment indicate amino acids that line the channel. Two amino acids at positions 74 and 75 form an extracellular gate (in pink and purple, respectively). Four cysteines (in yellow) contribute to two functionally important disulfide bonds. A red triangle denotes a caspase cleavage site located in the carboxyterminal domain. A box indicates a conserved carboxy-terminal segment of unknown significance. **b** Amino acids that line the inside of the channel are shown on one monomer of heptameric human PANX1 (PDB: 6LTO; aa 1-341^55^). The channel is oriented with the extracellular-facing side of the channel at the top. **c** The human PANX structure was used to model the extracellular gate of zebrafish Panx1a/Panx1b by substituting as required and repacking only amino acids 74/75 against a rigid backbone. As a control, human PANX1 was subjected to the same refinement and repacking protocol, creating a larger extracellular gate than the original cryo-EM structure. The diameters of the respective gates (in angstroms) are shown. **d** Molecular models of the extracellular gates of zebrafish Panx1a/Panx1b. Each protein bears one substitution relative to the human PANX1 structure. **e** An alanine was modeled to mimic a constitutively ATP-permeable state. Molecular graphics were produced with PyMOL v2.4.1 (Schrödinger, LLC).

As shown in Fig. 9a, a sequence alignment served as a starting point for a structural comparison between human PANX1 and the two zebrafish pannexins. A high degree of sequence identity is evident, especially throughout the amino acids that line the channel (Fig. 9b). Two pairs of disulfide-bonded cysteines in an extracellular facing domain of the channel are conserved in accordance with their established role in channel function^58^.

The pannexin channel has three gates, each corresponding to a point of restriction (Table 1). Among these gates, a sequence comparison of Panx1a and Panx1b with human PANX1 revealed that the first and most narrow gate had enough sequence diversity to be explored further by molecular modeling. In human PANX1, the extracellular gate is described by W74 and R75. The guanidino group of R75 is positioned to support a favorable cation-pi interaction with the indole ring of W74 and an ionic bond with D81. Either or both interactions may be necessary for the function of the gate.

**Table 1:** A comparison of gates between human PANX1 and zebrafish Panx1a/Panx1b.

	Human PANX1	zebrafish Panx1a	zebrafish Panx1b
Gate 1	W74-R75	W74-C75	M74-R75
Gate 2	I58	V58	V58
Gate 3	T21-E22-P23	S21-E22-N23	S21-D22-S23
CBX^a^ binding motif	I247-I258-F262	I247-V258-L262	L247-V258-V262

^a^Carbenoxolone (CBX).

To produce models of the Panx1a and Panx1b gates, the backbone of human PANX1 structure (PDB: 6LTO) was fixed in place, substitutions were made, and the side chains repacked. Before this was done, a control for the modeling study was performed by repacking the W74 and R75 side chains of human PANX1 according to the same protocol used to model the Panx1a and Panx1b extracellular gates. The rotamer sampling method favored a wider gate due to an edge-to-edge packing of the indole ring of W74 (Fig. 9c) versus a staggered packing observed in the original cryo-EM structure, increasing the diameter of the gate from 8.1 to 10.9 A. While the biological significance of these observed and modeled conformations is unknown, it demonstrates that side-chain motions may limit the size of the extracellular facing gate in the absence of backbone conformational changes.

In Panx1a, a cysteine (C75) replaces an arginine found in human PANX1 and Panx1b (Fig. 9d). While the loss of a positive charge and a nearby ionic bond could affect anion selection at this gate, a cysteine is still poised to support hydrophobic interactions with W74. In Panx1b, methionine (M74) substitutes for tryptophan, and the adjacent arginine (R75) is preserved. The modeled diameter of M74 was determined to 8.3 A, comparable to W74 in the staggered conformation. To explain the reduced ATP transit that we have observed during our physiological experiments for Panx1b, we speculate that a methionine substitution may sample different conformations that either favor the closed state of the gate or simply create a smaller gate and thereby less permeable to ATP.

A W74A substitution is associated with increased channel activity, increased ATP release, and a higher positive holding potential^57^. When we modeled an A74 variant of human PANX1, the diameter of Gate 1 was 11.2 A (Fig. 9e). The distance is comparable to the 10.9 A observed in wild-type (WT) PANX1 protein when the indole ring of W74 is in its most open edge-to-edge conformation. If the A74 variant extracellular facing gate is incapable of closing like the wild type protein, we question whether that is sufficient to explain the functional differences or if there is an alternate ATP-permeable state that the A74 variant is incapable of forming. Other amino acid substitutions at position 74 may be required to answer this question.

## Discussion

The roles of Panx1 channels in neuronal excitability and experimental epilepsy models are controversial and incompletely understood^59,60^. Here, the lower vertebrate zebrafish model was used to investigate the pro-convulsant activities of *Panx1*. Our results demonstrate a subfunctionalization of Panx1 ohnologs affecting seizure propensity. We identify the loss-of-function of the *panx1a* gene as the most discernable factor diminishing seizure susceptibility in gene-edited zebrafish by reducing extracellular ATP and affecting biological processes related to transport and metabolism. Furthermore, in both gene-edited larvae and after acute blocking of *Panx1* channels, extracellular ATP and *P2rx7* were identified as factors contributing to seizure susceptibility. However, the differences observed between chronic and acute blocking of *Panx1* channels advocate for caution when comparing genetic and pharmacological models directly.

To the best of our knowledge, there is no direct evidence for Panx1 being epileptogenic, and seizures are not known comorbidity in patients with loss-of-function or gain-of-function mutations in human PANX1^61–63^. However, support for Panx1 contributing to seizures is from increased human PANX1 protein expression and seizure activity found in epileptic tissue^9,12,13^. In rodents, activation of Panx1 augments aberrant bursting in the hippocampus and contributes to epileptiform seizure activities^64^; meanwhile, inactivation leads to a reduction^8–10,15^.

Here, blocking the gamma-aminobutyric acid (GABA)(A) receptor complex with PTZ evoked robust SLE as reported previously in zebrafish^36^ and mouse models^8^. The two Panx1 ohnologues show both pro- and anticonvulsant activities in the presence of PTZ. Similar opposing seizure activities in mouse Panx1 models are cell-type and brain region-specific^16^. The genotype-specific differences described here are best explained by the distinct expression localization and biophysical properties of Panx1a and Panx1b^19,20,30^. Like mammalian Panx1^18,34,35^, Panx1a is broadly expressed throughout the adult zebrafish^20,30^. Here, the detection of Panx1a in the neuropil of the OT and spinal cord of larvae, using a recently described antibody^32^, resembles the vesicular pattern of mouse Panx1 in axon bundles found in the olfactory and vomeronasal organs^53,65^. Previously, the expression of Panx1b was considered restricted to the nervous system in neurons and Muller glia of the adult retina^20,31^. In contrast, Panx1b immunoreactivity was detected in the OT and spinal cord of 6dpf larvae between densely packed neurons using the same antibody, which detects Panx1b in end-feet of Muller glial cells in the adult retina^39^.

The recent expansion of the E/I imbalance theory includes a recognition of an astrocytic basis of epilepsy in Mammalia. The theory is based on synaptic strength and plasticity modulation by astrocyte-released ATP acting on postsynaptic P2X receptors^66^ and basal extracellular adenosine levels regulation by an astrocytic enzyme adenosine kinase during seizure activity^67,68^. The role of astrocytes in seizures was demonstrated in mice with a GFAP-Cre driven cell-type specific deletion of Panx1^15^. In the zebrafish, results are inconclusive about astrocytic roles in epileptic seizures. Zebrafish lack GFAP-positive astrocytes in regions of interest but may contain GFAP-negative astrocyte-like cell types we presently have no definitive biomarker for. Further, the staining of GFAP-positive radial glial cells in the spinal cord did not provide compelling evidence for Panx1a or Panx1b expression in this cell type. To the best of present knowledge, equally exciting alternatives exist; the recruitment of GFAP-positive astrocytes to epileptic seizures is specific for Mammalia and developed when the relationship between neurons and astrocytes became more complex, or an uncharacterized GFAP-negative astrocyte-like cell population contributes to seizures in zebrafish alongside neurons.

Hypotheses regarding Panx1s’ involvement in epileptic seizures vary, with evidence pointing toward the ability to release the neurotransmitter ATP^9,10,69^. Reports show extracellular ATP is reduced in brain tissue preparations deriving from mice with global loss of Panx1^10,53,65^, or astrocytic deletion of Panx1^15^, and extracellular ATP concentrations increase during high levels of neuronal activity and SLE^70,71^. Since extracellular ATP levels depend on the rate of cellular release and enzymatic degradation, purinergic signaling, or the ratio between ATP and its metabolites^70,71^, we quantified extracellular ATP and the expression of genes involved in the process. In line with observations in mice, zebrafish lacking Panx1a channels show reduced ATP release efficiency, extending previously reported differences in channel activation kinetics and open times^19,20^. Furthermore, RNA-seq analysis and quantification of selected biomarkers provided evidence of how ATP-related mechanism(s) contribute to pro-convulsant activities of Panx1a. The enriched biological processes and coordinated expression changes aligned with the opposite ATP release activities of *panx1a*^−*/−*^ and *panx1b*^*−/−*^ larvae, with DKO larvae representing mixed or bi-directional trends. The opposing trends of the transcriptome and ATP release might mirror how neuronal circuits are impacted by the distinct biophysical properties of Panx1 channels^20^. We recently reported that Panx1b under “normal” conditions modulates the circadian clock system, suggesting similar roles in modulating metabolism and sleep/wakefulness in fish and rodents^31,72^. Since epilepsy is one of the conditions in which symptoms often worsen at a particular time of the day, it will be vital to investigate this connection in the future.

The recent description of Panx1 structures^54,56,73^ allowed us to investigate the relationship between the zebrafish Panx1 ohnologues and the human paralog by structural modeling. Previous work by the Dahl group identified that ATP permeates^74^ and activates mammalian Panx1 channels^75^ and that amino acids W74 and R75 play a critical role in the process^76^. Notable structural similarities and differences exist in the pore region of Panx1a, Panx1b, and human PANX1. However, the conservation of W74 at a critical gate in both human PANX1 and Panx1a suggests similar permeability of ATP in an open state. We propose that this similarity enables Panx1a, like its mammalian paralogs, to contribute to pro-convulsant activity via ATP signaling, as ATP release capabilities are reduced in the panx1a^−*/*−^ larvae after PROB treatment and in the DKOs. In turn, the lack of the conserved W74 could render Panx1b less competent for ATP signaling and seizure-like activities.

Functional interaction of the mammalian P2x7R-Panx1 complex is established^40^. Moreover, like Panx1, mammalian P2x7R expression is upregulated in seizure conditions^77^, and targeting P2x7R in mice reduces epileptic seizures in some but not all models^78–81^. Here, the downregulation of P*2rx7* in *panx1a*^−*/−*^ larvae^32^ coincides with a significant reduction of SLEs. However, when P2rx7 receptors were blocked in TL, and *panx1b*^−*/−*^ larvae, only a moderate reduction of SLEs was observed, advocating for Panx1a and not P2rx7 as the principal driver of seizure-like activities.

This study has taken an important step toward establishing Panx1 zebrafish models for studies of seizure mechanisms. We anticipate that the unique possibilities afforded by this behaving lower vertebrate model will play crucial roles in dissecting the mechanisms in vivo of how the brain can be guarded against epileptic seizures and which processes require Panx1.

## Methods

### Zebrafish lines

All zebrafish (*Danio Rerio*) of strain Tubingen long fin (TL) were maintained in groups with mixed sex in a recirculation system (Aquaneering Inc., San Diego, CA) at 28 °C on a 14 h light/10 h dark cycle. All animal work was performed at York University’s zebrafish vivarium and in S2 biosafety laboratory following the Canadian Council for Animal Care guidelines after approval of the study protocol by the York University Animal Care Committee (GZ#2019-7-R2). The *panx1* mutant lines were generated and characterized in-house^32,39^.

### TALEN constructs

The TALEN constructs were synthesized in Dr. Stephen Ekker’s lab (Mayo Clinic Cancer Center, Rochester, MN). Briefly, TALEN assemblies of the RVD-containing repeats were conducted using the Golden Gate approach^82^. Once assembled, the TALE repeats were cloned in the pT3TS-GoldyTALEN expression vector^83,84^. TALEN expression vectors were linearized with the *Sac*I restriction endonuclease (ThermoFisher Scientific, Canada) for 15 min at 37 °C, and used as templates for in vitro transcription. Capped cRNAs were synthesized from TALEN pairs mixed 1:1 using the mMESSAGE mMACHINE T3 Transcription kit (Life Technologies, Canada). The mixture of the two TALEN cRNAs was purified using the Oligotex mRNA Mini Kit (Qiagen Inc., Toronto, Canada). TALEN cRNAs were diluted in DNase/RNase-free water (Life Technologies) to the final concentration of 1 µg/μL and stored at −80 °C before microinjection.

### Microinjection & genotyping

One-cell stage zebrafish embryos were injected with TALEN cRNAs pair at doses ranging from 30 to 100 pg/nl. The toxicity of the injected cRNAs was determined at 24 h post fertilization (1dpf) by calculating the proportion of healthy, dead, and malformed embryos at each dose. The condition resulting in more than 50% post-injection survival was selected for further injections. Genomic DNA (gDNA) was extracted from groups of ten injected embryos 4 days post-fertilization (4dpf) to examine the TALEN mutagenesis efficiency. Briefly, the individual larva was incubated in 100 mM NaOH at 95 °C for 15 min. After cooling to room temperature, the one-tenth volume of 1 M Tris (pH8.0) was added to the extracts to neutralize the NaOH^85^. Finally, 1 volume TE buffer pH8.0 was added, and gDNAs were stored at −20 °C. A small indel mutation screen used PCR followed by *AfeI* (*panx1a*) and HindIII (*panx1b*) restriction enzyme (RE) digests. Indel mutations were confirmed by sequencing (Eurofins Genomics LLC, KY, USA) of gel-purified PCR products cloned into the pJet1.2 cloning vector (Life Technologies).

### Generation of knockout lines

Adult mosaic zebrafish (F0) were anesthetized in pH-buffered 0.2 mg/ml ethyl3-aminobenzoate methane sulfonate solution (MS-222, Sigma-Aldrich). The caudal fin (2 mm of the end) was removed using dissecting scissors (WPI Inc., FL, USA) and placed into 1.5 ml collecting tubes. The fin gDNA was isolated and screened for indel mutations as described^83^. Adult F0 zebrafish with desired indel mutations in the *panx1* genes were out-crossed to WT TL zebrafish. F1 offspring were analyzed by PCR and *AfeI/HindIII* digestions to verify germline transmission of mutations. Heterozygous F1 mutants were in-crossed to establish homozygous F2 mutants. Homozygous *panx1a*^−/−^ and *panx1b*^−/−^ lines were in-crossed to generate a DKO line. All experiments described were performed with progenies of >F4 generations. Later generations were routinely tested for the identity of the genotype.

### Immunohistochemistry

Zebrafish larvae (6dpf) were humanely euthanized, fixed in 4% paraformaldehyde (PFA) in 1xPBS overnight at 4 °C, followed by cryoprotection in 30% sucrose in 1xPBS. After embedding in Tissue-Tek O.C.T compound frontal 15 µm sections were cut on a cryotome (Thermofisher). Samples were washed three times for 5 min with 1×PBS containing 0.1% Tween-20 (PBST) at RT. Unspecific binding sites were blocked with freshly prepared 5% normal goat serum (NGS, Sigma-Aldrich) in PBST for 1 h at 4 °C. Following blocking, samples were incubated with primary antibodies (all 1:100, affinity-purified rabbit anti-*Panx1a*, code#ZOI-A2, affinity-purified rabbit anti-*Panx1b*, code#ZOI-B2; both Davids Biotechnologie GmbH; 1:100, rabbit anti-GFAP, code AB5804, Sigma-Aldrich) overnight at 4 °C. The specificity of the custom-made antibodies has been demonstrated using the *panx1a*^*−/−*^ and *panx1b*^*−/−*^ fish lines used in this study^31,32^. Subsequent washes with PBST were for 1 h at 4 °C. Alexa 488 and Alexa 546 goat anti-rabbit/mouse secondary antibodies (1:3000 in 1% NGS PBST, Life Technologies) were applied for 1 h at RT$.$ After three washes with PBST followed by one wash with PBS, specimens were mounted on microscope slides using ProLong Antifade with DAPI (Thermofisher). Confocal images were collected using LSM-ZEN2 software (Zeiss LSM700 system; Carl Zeiss MicroImaging, Oberkochen, Germany) with a Plan-Apochromat 20×/0.8 or Plan-Apochromat 63×/1.3 oil DIC M27 objectives. The software optimized pixel resolution, line averaging, gain, and digital offset. Post image collection composite figures were created using Adobe Photoshop 2021.

### RNA extraction and RT-qPCR

Total RNAs were extracted from three independent pools of ≈30 7dpf larvae using RNeasy Plus Mini Kit (Qiagen). The iScript Reverse Transcription Supermix (Bio-Rad Laboratories, Mississauga, Canada) was used to reverse transcribe 1 µg of total RNA. The cDNA equivalent of ≈133 ng total RNA was analyzed in triplicate by quantitative Real Time-PCR using the SsoAdvanced SybrGreen PCR mix and a CFX96 touch instrument (Bio-Rad). Cycling conditions: Polymerase activation and cDNA denaturation (one cycle: 98 °C for 30 s); 40 cycles of denaturation for 10 s at 98 °C; 20 s annealing/extension at 60 °C. All experiments included a melt curve analysis of PCR amplicons generated by ramping from 65 to 95 °C in 0.5 °C increments for 5 s/step. Raw cycle threshold values (Ct-values) were exported from the CFX Manager Software (Bio-Rad, Canada), and the relative gene expression was calculated using the Relative Expression Software Tool (REST-2009)^86^. The statistical significance was tested by a Pair Wise Fixed Reallocation Randomization Test^©^. Gene information and primer sequences are listed in Supplementary Table 4. The size of amplification products was <150 bp.

### RNA-seq analysis

The transcriptomes of all zebrafish lines used in this research were analyzed by RNA-seq (NGS-Facility, The Center for Applied Genomics, SickKids, Toronto, ON). The data was derived from sequencing three independent pools of ≈30 age-matched larvae (6dpf). The RNA library preparation was performed following the NEB NEBNext Ultra II Directional RNA Library Preparation protocol (New England Biolabs Inc., Ipswich, MA, USA). RNA libraries were loaded on a Bioanalyzer 2100 DNA High Sensitivity chip (Agilent Technologies) to check for size, quantified by qPCR using the Kapa Library Quantification Illumina/ABI Prism Kit protocol (KAPA Biosystems, Wilmington, MA, USA). Pooled libraries were paired-end sequenced on a High Throughput Run Mode flow cell with the V4 sequencing chemistry on an Illumina HiSeq 2500 platform (Illumina, Inc., San Diego, CA) following Illumina’s recommended protocol to generate paired-end reads of 126-bases in length. The post-sequencing processing to final read counts, normalization, and differential gene expression analysis used multiple software packages, including RSEM version 1.3.3 (http://deweylab.github.io/RSEM/) bowtie2 version bowtie/2.3.4.2 (http://bowtie-bio.sourceforge.net/index.shtml) to estimate the expression level of each sample. RSEM reports read counts, estimated lengths, and FPKM for each sample for each transcript and gene. For differential expression analysis, estimated read counts from RSEM output were compiled for each transcript. This transcript expression matrix was supplied to DESeq2 (https://bioconductor.org/packages/release/bioc/html/DESeq2.html) v.1.22.2 to detect differentially expressed transcripts. Filtering of the low expressed transcripts to increase power was automatically applied via independent filtering on the mean of normalized counts within the DESeq results() function. Note: The filtered transcripts have padj (FDR) value of “NA”. The final output of the DESeq2 results included TranscriptID, GeneID – Ensembl IDs; GeneVersion, GeneName, GeneBiotype, baseMean - Mean of normalized counts for all samples; log2FoldChange - Log2 fold change*;* lfcSE - Standard error; stat - Wald statistic; *P* value - Wald test *P* value; padj - Benjamini+ Hochberg multiple testing; BH adjusted *P* values.

### Transcriptome analysis

Genes that were significantly regulated according to the padjusted value (<0.05) were organized by up (>1) and down (<−1) regulation based on the associated logFC value. Gene ontology (GO) enrichment of biological processes from the FishEnrichR database was established after generating two gene lists (up-, downregulated) for each genotype (Supplementary Data 1). Fisher’s exact test with testing for the false discovery rate was used for correction. Biological processes related to the nervous system and *panx1* were grouped into broader GO terms for analysis and presentation. Clustergrams were produced for differential expression analysis using curated gene lists (Supplementary Data 2) and procedures implemented in the Matlab2019b Bioinformatics toolbox using Euclidean distance for hierarchal clustering and presentation as colored heatmaps.

### In vivo electrophysiology

Published procedures were used to prepare anesthetized 7 days post fertilization (7dpf) zebrafish larvae for in vivo electrophysiology^32,41^. Zebrafish larvae 7dpf were briefly anesthetized using 0.3 mM Pancuronium bromide (Sigma-Aldrich) for 2–3 min until the touch response stopped. Anesthetized larvae were immobilized in freshly prepared 2% low melting temperature agarose. An Olympus dissecting microscope was used to orient the dorsal aspect of the larvae to the gel surface. Embedded larvae were placed on the upright stage of an Olympus BX51 fluorescence microscope. 1 mL of egg water (E3; pH 7.2–7.4) was applied to the agar topically. Under direct visual guidance, a glass microelectrode (1.2 mM OD, ~1 µM tip diameter, 2–7 MΩ), backloaded with 2 M NaCl, was placed into the right OT. Local field potentials (LFP) were recorded using a Multiclamp 700B amplifier (Axon Instruments, San Jose, CA, USA). Voltage recordings were low-pass filtered at 1 kHz (−3 dB; eight-pole Bessel), high-pass filtered at 0.1 Hz, digitized 10 kHz using a Digidata 1550 A A/D interface, and stored on a PC running pClamp11 software (all Axon Instruments). The basal activity was recorded for 10 min under Light-ON conditions (1000 lux), during which images of the electrode placement were taken for reference. Then, 1 mL of E3 containing PTZ, for a final concentration of 15 mM, was added topically to the agar and recorded for an hour. Seizure activity was normalized to its baseline activity for each fish to account for the biological variability of individual brains. For drug testing, zebrafish larvae were exposed to treatment drugs, 75 µM PROB or 5 mM VPA, at the start of baseline recordings 10 min prior to the application of PTZ.

### Event detection and power spectral density

Seizure-like event identification was defined using the TL population as high-frequency events with large amplitudes (three times the standard deviation of the baseline activity determined by the algorithm), polyspikes, and a duration of 3 s or greater. If events did not meet these criteria, like in the DKO population, they were not classified as seizure-like. Inter-ictal-like events were identified as high-frequency events, with shorter amplitude compared to ictal-like events but greater than baseline activity (minimum 1.5 times the standard deviation of baseline) and shorter in duration (1–3 s). Event detection was automated using custom-developed codes in Matlab R2019b^87,88^. Events were quantified for comparison across genotypes and measured for significance using the Mann–Whitney test. The significance of the % of fish seized was determined using the Barnard exact test.

Welch’s method performed power spectral density estimation for baseline and PTZ recordings. A moving window for fast Fourier transform computation was used, and all windows were averaged. Changes in delta power were determined using the area under the power spectrum between 1 and 4 Hz. The trapz function in Matlab R2019b was used for calculation. PSD was measured for significance using a Kruskal–Wallis (between genotypes) or a Wilcoxon matched-pairs signed-rank test (between treatments) and presented as the mean ± s.e.m.

### Zebrafish locomotion assays

The Zebrabox behavior system and the ZebraLab analytical suite (ViewPoint Life Technology, Lyon, France) were used for automated extraction of behavioral outputs and video tracking. Tracking videos were recorded at 30 frames per second (fps) under infrared light illumination using a Point Gray Research Dragonfly2 DR2-HIBW. A lightbox provided visible light for recordings at 30% light intensity. 7dpf larvae were observed in clear 96-well plates maintained at 28 °C. Locomotor activity scores (Δpixel ± s.e.m.; *n* = 36 per group) were analyzed for larvae’s resting (25 min), baseline (30 min), and PTZ-induced behaviors (1 h) using two-way repeated-measures ANOVA with the Greenhouse-Geisser correction followed by a Bonferroni multiple comparisons test. PTZ was administered in egg water at 15 mM final concentration. In pharmacologically treated TL, PROB (75 µM; *n* = 36), VPA (5 mM; *n* = 60 per group), and A-438079 hydrochloride hydrate (A43; 100 µM; *n* = 46) were administered in egg water 30 min prior to PTZ. Concentrations for PROB^89^, VPA^36^, and A-438079^90^ were chosen from the literature. A PROB dose-response test can be found here (*n* = 18; Supplementary Fig. 7). The Areas Under the Curve (AUCs) were determined from activity plots and analyzed by one-way ANOVA followed by a Tukey’s multiple comparison test via an unpaired *t*-test or a Mann–Whitney test.

### Stage II and III seizure-associated behavior scoring

Videos extracted from the ZebraLab software were used for manually and blindly scoring stages II and III of seizure-associated locomotor behavior described in literature^36^. Stage II manifests as a ‘whirlpool-like’ rapid circling the well (Supplementary Movie 1) and stage III as a clonic-like convulsion followed by a loss of movement and posture (Supplementary Movie 2). Stages II and III were scored at 2 min intervals for 1 h after the PTZ application. Stage I, described as excessive hyperactivity, was not scored due to ambiguity and lack of quantifiable features for this stage; however, it is represented in the first 10 min of the activity plots as a sharp increase in locomotor activity.

### Self-organizing maps for behavioral phenotyping

An unsupervised machine learning technique, Self-Organizing Map (SOM), was used to examine behavioral similarities between TL and *panx1* knockouts in response to 15 mM PTZ treatment for an hour. MathWorks^®^ Deep Learning Toolbox function ‘selforgmap’ (MathWorks, MATLAB R2020a) was applied to eight behavioral outputs extracted from the ZebraLab software; activity score (∆pixel), total duration of movement (total dur; sec), freezing behavior count and duration (freeze count, freeze dur; 0 mm/sec), normal swimming behavior (mid count, mid dur; 0–20 mm/sec) and burst (burst count, burst dur; >20 mm/sec). The SOM neural network was trained on 8640 data points for each behavioral parameter (4 genotypes × 36 larvae per genotype × 60 time points for PTZ response). The SOMs were constructed to contain 16 cluster centers positioned hexagonally in a 4-by-4 two-dimensional map. Distances between cluster centers (‘linkdist’) determined a representative center for each larva’s data input vector. The cluster center positions were updated to an average position of all the input vectors for which they were the representative centers. The network was trained on a batch algorithm, by presenting all the data at once to the network and training it in 200 iterations. The process classified each larva to a representative center based on most of its behavioral data clustering at the center. Fisher’s exact tests were performed for individual behavioral outputs, comparing each genotype’s larvae distribution across all cluster centers. The absolute difference in the number of classified larvae between genotypes for each cluster was averaged to acquire heatmaps of clustering similarity; smaller values represented higher similarity. Behavioral data of A-438079 (*n* = 46) and PTZ treated TL larvae was applied to the trained network to assess the behavioral similarities between pharmacological treatments and genetic targeting of Panx1.

### Zebrafish larvae survival assessment

7dpf TL and *panx1* knockout larvae (*n* = 80 per group) were assessed for survival under brightfield microscopy every hour for 10 h and once at 24 h after the application of PTZ. The assessment included examining for circulation/heartbeat and body movement and degradation. Group survival rates were analyzed with the log-rank (Mantel–Cox) test and plotted as Kaplan–Meier curves.

### Extracellular ATP assay

Fifty larvae (7dpf) were collected to quantify ATP and protein at experimental endpoints. Larvae were homogenized in 500 µl ice-cold of Phosphate-Buffered Saline containing 100 µM ARL-67156 (Sigma-Aldrich) and Halt Protease inhibitor (Thermo-Scientific) (1:100) for 1 min at 30 Hz using the Tissuelyser^LT^ (Qiagen). Homogenates were transferred to chilled Eppendorf tubes (−20 °C) and centrifuged at 12,000 *rpm* for 2 min. Supernatants were collected and snap-frozen in liquid nitrogen before storage at −80 °C. Extracellular ATP measurements used a 96-well format (Greiner Bio-One) and the Molecular PROBes^®^ ATP determination Kit described by the manufacturer (Life Technologies). ATP was quantified in replicates of 6 using the Synergy H4 Hybrid Multi-well Plate Reader (Biotek)^65^. Luminescent assay parameters used: plate temperature set to 28 °C; a low-intensity shake of the plate for 3 s prior to reading; a 5 s integration time per well; gain set at 150. The ATP concentration in experimental samples was calculated from ATP standard curves (0–1 µM ATP) included in the same 96-well plate. Data were exported from the Gen5 Data Analysis Software (Biotek) and analyzed in Excel and Matlab. The estimation plotting feature of Graphpad was used to test for statistical significance between treatment groups. ATP was represented as a normalized concentration per mg of protein. A NanoDrop Spectrophotometer measured protein content in 2 µL of supernatant from homogenized samples. The final ATP content was expressed concerning the amount of protein content in each pooled larvae sample after we demonstrated that the weight of larvae and homogenate protein content was linearly correlated (Supplementary Fig. 8).

### Molecular modeling

The sequences of zebrafish Panx1a (Uniprot: Q7ZUN0) and Panx1b (Uniprot: F1QSR7) were aligned initially with Clustal Omega^91^ to human PANX1 (Uniprot: Q96RD7). Using the alignment as a guide, zebrafish Panx1a and Panx1b were then threaded into the cryo-EM structure of human PANX1 (PDB: 6LTO) using SWISS-MODEL^92^. Using the FixBB module of Rosetta v3.12^93^, the side chains of amino acids 74 and 75 were altered and repacked following the standard rotamer selection parameters of the program. Non-crystallographic symmetry restraints were included to produce a heptameric assembly. The diameter of the extracellular gate was measured with PoreWalker^94^.

### Pharmacology

All chemicals were purchased from Sigma-Aldrich (Mississauga, Canada): Pentylenetetrazole (PTZ; 15 mM; cat#P6500), Pancuronium bromide (Panc; 300 µM; cat#P1918), probenecid (PROB; 75 µM; cat#P8761), Valproic Acid (VPA; 5 mM; cat# P4543) and A-438079 hydrochloride hydrate (A43; 100 µM; cat#A9736). All concentrations referred to in the results are final.

### Statistics and reproducibility

All statistical analyses were performed in Matlab R2019b or in GraphPad Prism 9. Results are represented as the mean ± standard error of the mean (s.e.m.). A minimum of *n* ≥ 3 independent experimental replicates for each analysis were generated. A *P* value < 0.05 was considered statistically significant. G*Power analysis was used to confirm the required sample size and the statistical power of the acquired data^95^. The figure legends indicate sample sizes, statistical tests, and *P* values for all experiments. Further statistical details are disclosed in Supplementary Data 3.

### Reporting summary

Further information on research design is available in the Nature Research Reporting Summary linked to this article.

## Supplementary information

Supplementary Information

Description of Additional Supplementary Files

Supplementary Data 1

Supplementary Data 2

Supplementary Data 3

Supplementary Data 4

Supplementary Movie 1

Supplementary Movie 2

Reporting Summary

## Data Availability

Data generated or analyzed during this study are included in this published paper and Supplementary Data 3. Source data underlying the main figures are presented in Supplementary Data 4. The RNA-seq data are deposited in the Gene Expression Omnibus (GEO) repository (https://www.ncbi.nlm.nih.gov/geo/). The database record is GSE181853. Any remaining information can be obtained from the corresponding author upon reasonable request.
