# Supplementary material for: Panx1 channels promote both anti- and pro-seizure-like activities in the zebrafish via p2rx7 receptors and ATP signaling
Source: Commun Biol. 2022 May 18;5:472. doi: 10.1038/s42003-022-03356-2 (PMC9117279; doi:10.1038/s42003-022-03356-2)
Supplement: Supplementary file 1 — Supplementary Information [file 42003_2022_3356_MOESM1_ESM.pdf]

Panx1 channels promote both anti- and pro-seizure-like activities in the zebrafish via *p2rx7* receptors and ATP signaling

Paige Whyte-Fagundes[1,2,3], Daria Taskina[1,2], Nickie Safarian[1,2], Christiane Zoidl[1,2], Peter L Carlen[3,4], Logan W Donaldson[1], Georg R Zoidl[1,2,3*]

[1] Department of Biology, York University; Toronto, Ontario, M3J1P3; Canada
[2] Center of Vision Research (CVR), York University; Toronto, Ontario, M3J1P3; Canada
[3] Krembil Research Institute, University Health Network, 60 Leonard Ave, Toronto, ON M5T 1M8, Canada
[4] Department of Medicine, Physiology and BME, University of Toronto, 399 Bathurst St., 5w442, Toronto, ON M5T 2S8, Canada

Supplementary Information (available in this document)

Supplementary Figures 1–8

Supplementary Tables 1–4

Supplementary Movies 1,2

Supplementary Figures

Supplementary Figure 1: *The differential localization of Panx1a and Panx1b in the zebrafish larval diencephalon and spinal cord.*

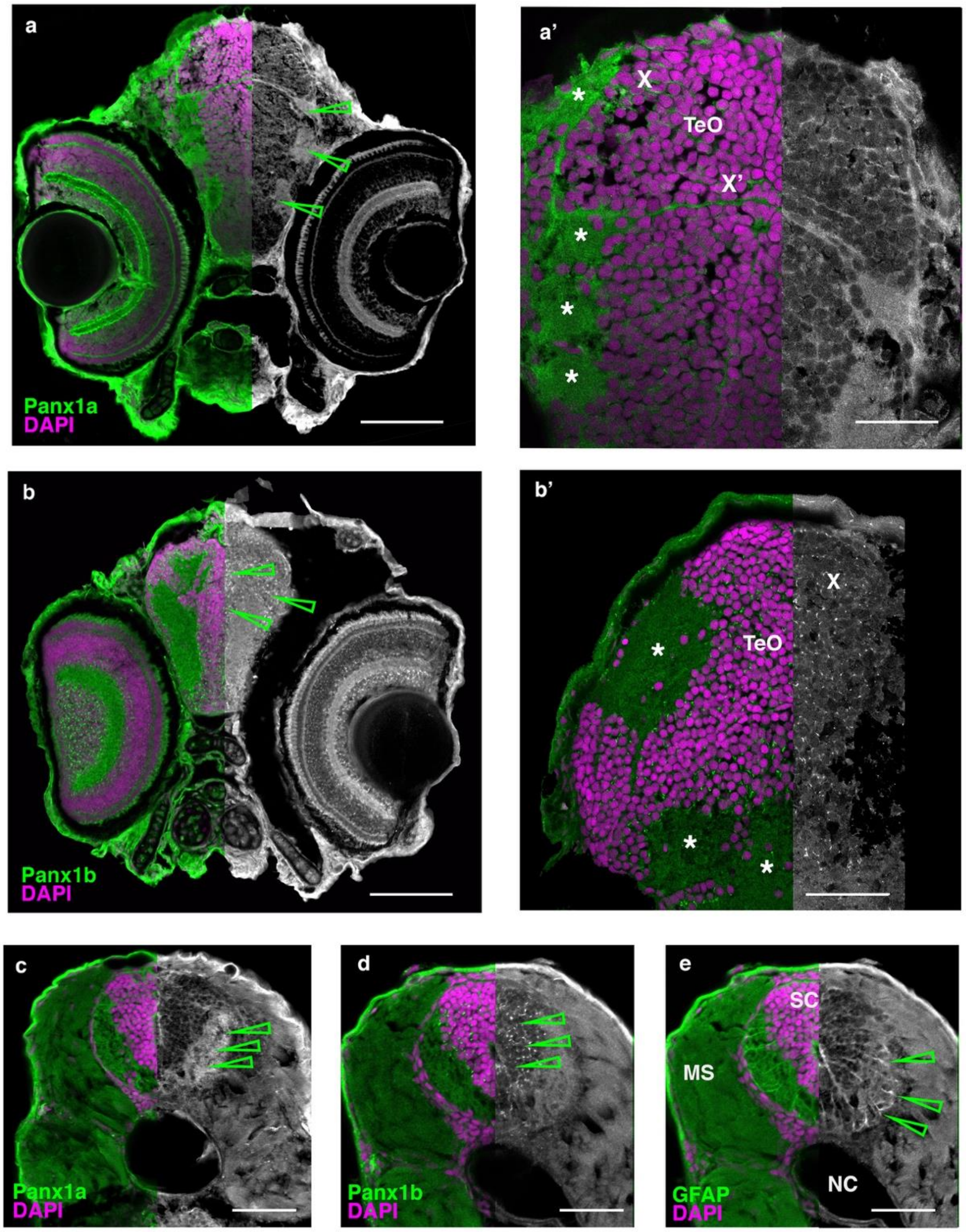

a) Panx1a immunoreactivity was detected in the neuropil of arborization fields (AFs) in the diencephalon's retinorecipient areas (open arrows). **a')** shows Panx1a immunoreactive AFs (stars) and a meshwork of potential fiber bundles (X) penetrating layers of densely packed neuronal cells in the optic tectum (TeO), or a commissural fiber between hemispheres (X'). **b)** Panx1b

immunoreactivity was dotted and localized in densely packed neuronal cells layers. **b'**) shows part of the TeO with a region of Panx1b immunoreactivity (X) adjacent to AFs (stars). Note the non-overlapping localization of Panx1a and Panx1b in the retina of 6dpf larvae in both a) & b). **c)** Like in the diencephalon, Panx1a immunoreactivity was found in the neuropil of the spinal cord (arrows), or **d)** focal spots between densely packed neuronal cells in the optic tectum (Panx1b, arrows). **e)** The localization GFAP-positive radial glial cells in frontal sections of the spinal cord. Arrows point at the end-feet of astrocytes. Abbreviations: TeO, tectum opticum, MS, muscle, NC, notochord, SC, spinal cord, GFAP, Glial fibrillary acidic protein, DAPI, 4′,6-diamidino-2-phenylindole. Scale bars a,b 100µm; a' 30µm; b' 20µm; c-e 25µm.

Supplementary Figure 2: *One hour baseline recordings of untreated larvae.*

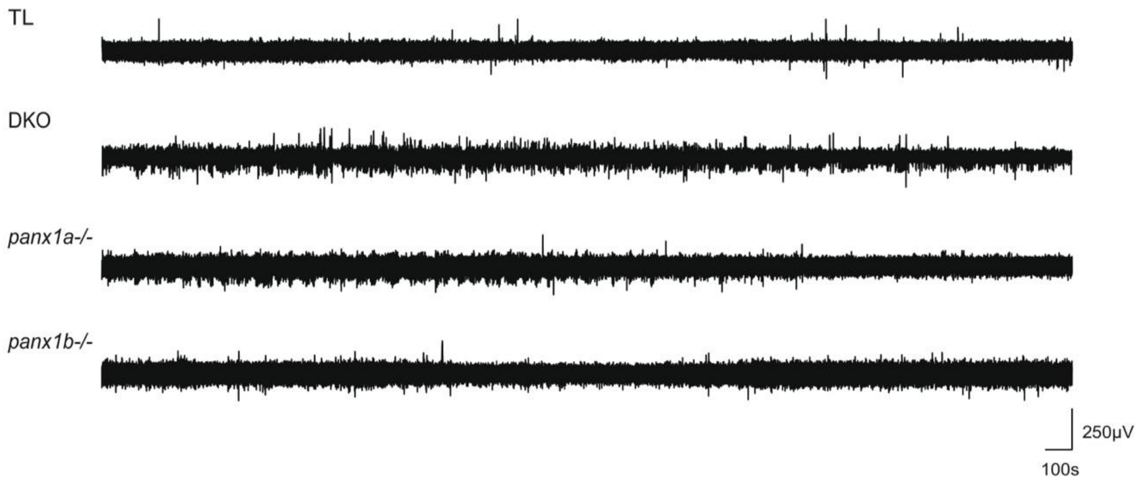

Supplementary Figure 3 *Comparison of PTZ treatment to baseline activity is indistinguishable*.

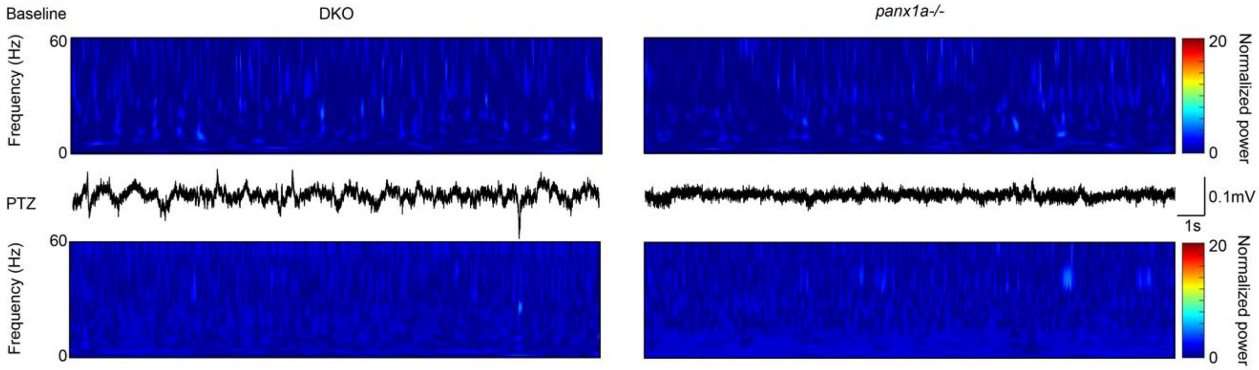

Spectrograms of baseline activity (top) directly compared to recordings during PTZ treatment (bottom) for DKO (left) and *panx1a*$^{-/-}$ (right) show that PTZ treatment does not affect neural

activity in these genotypes. Sample traces from PTZ recordings at the 1hour mark are included for reference, revealing no major spiking activity.

Supplementary Figure 4 *Self-Organizing Maps (SOM) emphasize phenotypic similarities of PTZ treatment in TL and panx1 knockouts.*

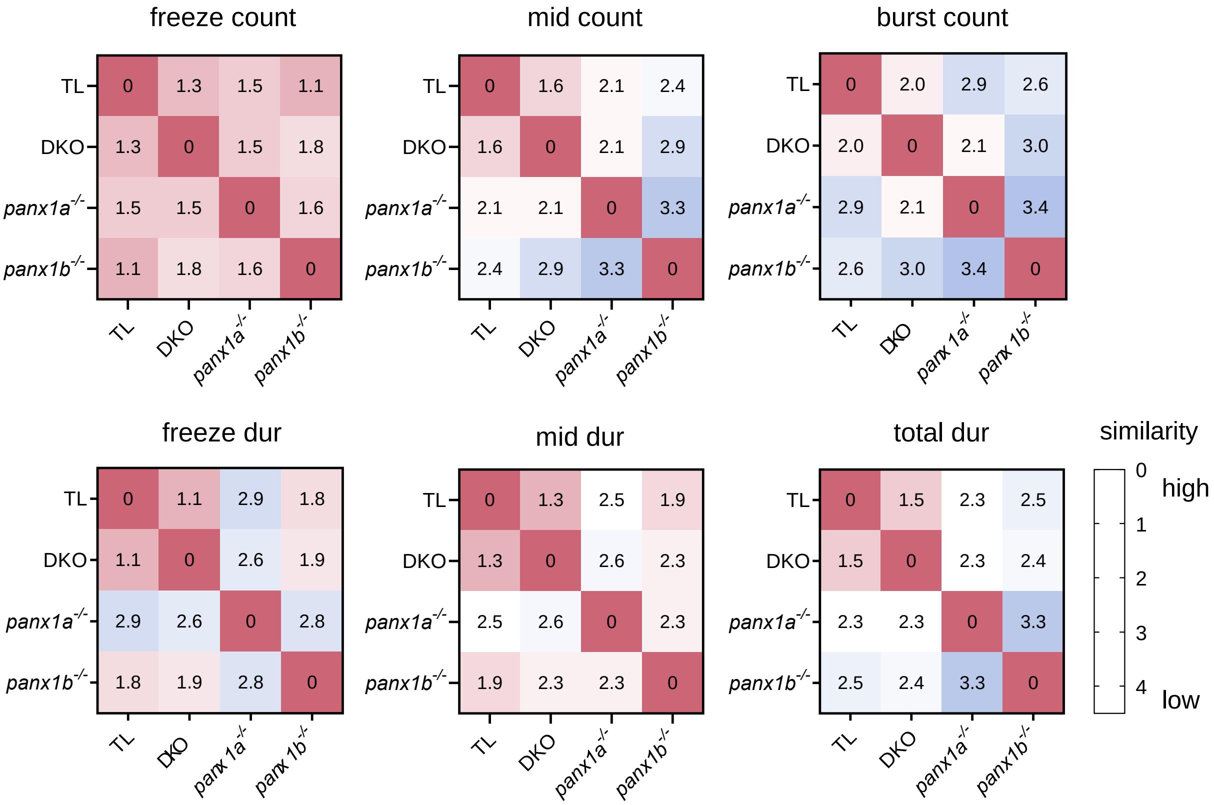

SOM was applied on behavioral outputs extracted from the ZebraLab software on PTZ treated TL and *panx1* knockout larvae (n = 36 per group). The absolute difference between the number of TL and *panx1* knockout larvae assigned to each cluster in the SOM were averaged for all clusters and visualized as relative similarity in a form of heatmaps. Smaller value and warmer color represent higher similarity. TL and DKO exhibited the most similar PTZ-induced phenotypes across freezing and normal locomotor behaviors. In general, *Panx1a*$^{-/-}$ and *panx1b*$^{-/-}$ were most distinct from one another, particularly for freezing duration, normal behavior counts and total duration of movement ($P < 0.0001$). Overall freezing and normal behaviors showed less variation among genotypes compared to general activity and bursting behaviors. See Supplementary Data 3 for details on statistical significance for each behavioral output and genotype comparison.

Supplementary Figure 5 *Comparison of TL with and without PROB treatment only.*

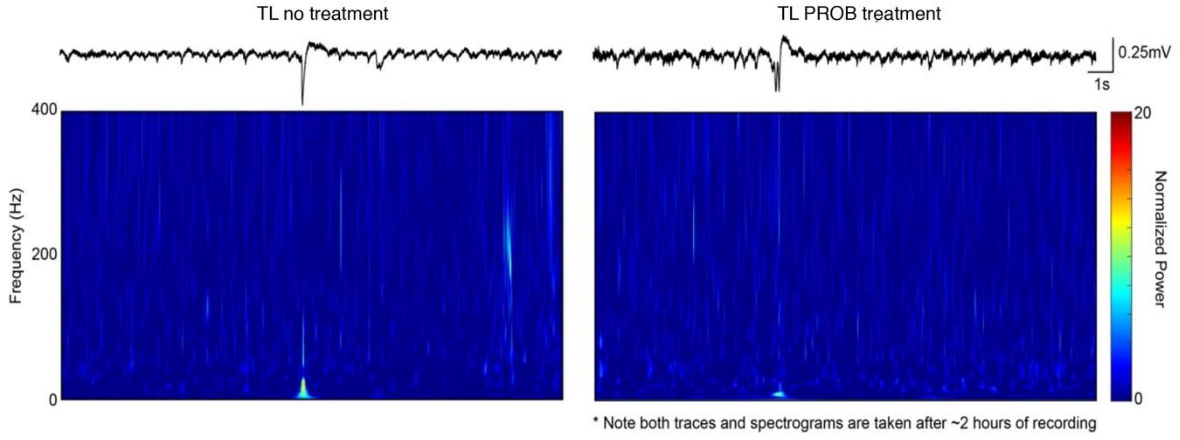

Spectrograms of baseline activity taken after 2hours of recording from TLs with no drug treatment (left) at all and TLs treated with probenecid only (right). This comparison shows that PROB is not inducing a toxicity effect to prevent seizure-like events from occurring as these spectrograms look like baseline activity.

Supplementary Figure 6 *Venn diagrams of differentially regulated genes and biological processes.*

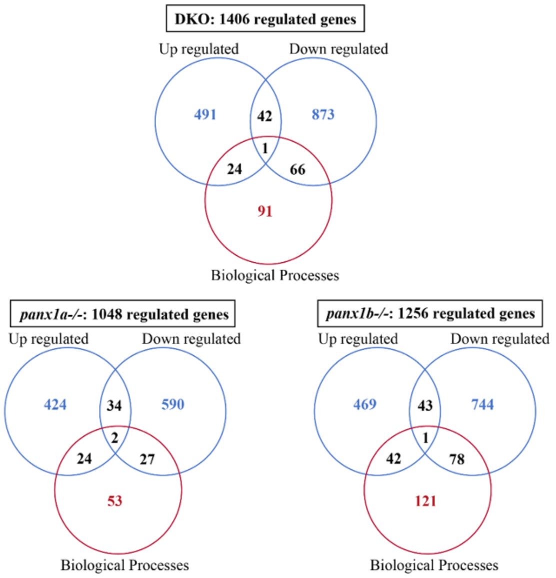

Venn diagrams highlighting how many genes are regulated in each of our *panx1* genotypes and show how many genes fit into biological processes according to FishEnrichR ontology database. They also reveal the overlap in regulated genes.

Supplementary Figure 7 *Concentration dependent reduction of PTZ-related hyperactivity in TL larvae by Probenecid.*

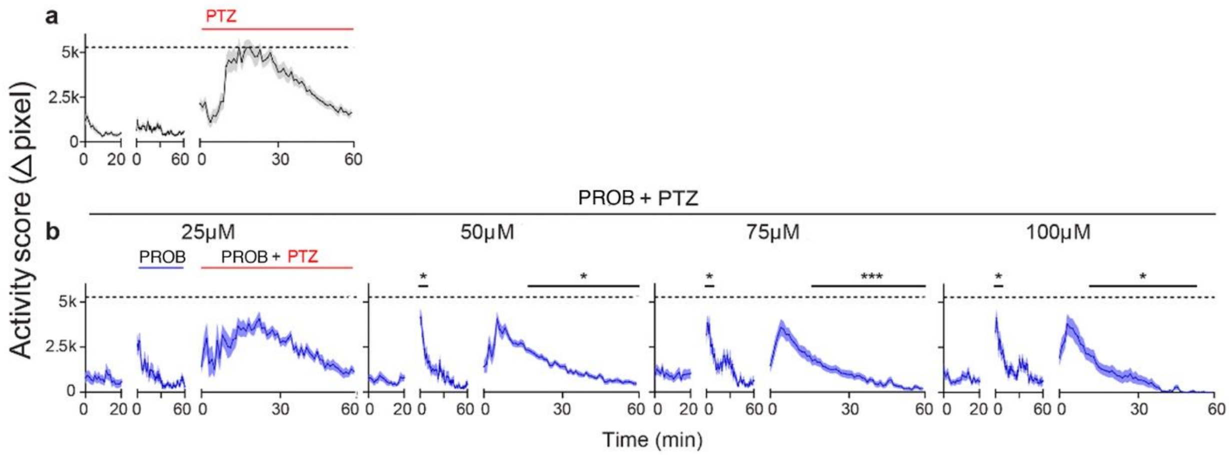

a) 7dpf TL larvae (n = 30) were treated with 15mM PTZ, or **b)** incubated with 25µM, 50µM, 75µM, and 100µM Probenecid (PROB; n = 18 per concentration) one hour before treatment with PTZ. PROB significantly increased activity (Δpixel; mean ± s.e.m.) compared to baseline, and significantly reduced PTZ-induced activity above 50µM. PROB applied at 75µM reduced PTZ-induced activity, without the evidence of substantial toxicity (activity level 0 within one hour). Dashed lines indicate max average activity for PTZ treated TL. *P < 0.05, ***P < 0.001.

Supplementary Figure 8 *Linear correlation of larval weight and protein concentration*.

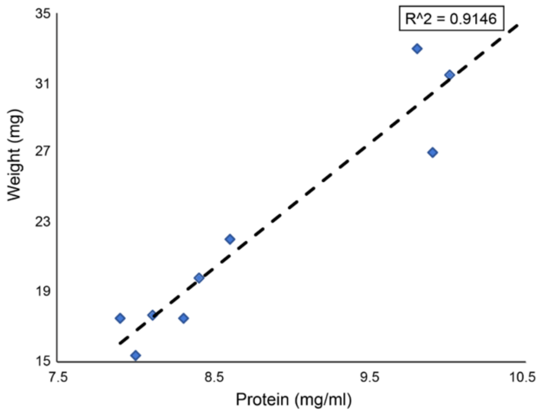

Plotting the weight of pooled larvae (n = 50 larvae/sample) in mg against the amount of protein in mg/ml that was measured from the supernatant of the homogenate with a spectrophotometer demonstrates a clear linear relationship ($R^2 = 0.9146$). Therefore, ATP concentrations were plotted per protein content to account for biological variance of larval weight.

Supplementary Tables

Supplementary Table 1 RT-qPCR values for Immediate Early Gene (IEG) regulation in TL and *panx1* knockout larvae treated with PTZ for one hour (**Figure 4f**). Expression values, respective to non-treated controls, are >1 for upregulated IEGs and <1 for downregulated IEGs. *P*-values in bold = P < 0.05.

**Gene**	**TL**	*P*-value	**DKO**	*P*-value	***panx1a***$^{-/-}$	*P*-value	***panx1b***$^{-/-}$	*P*-value
**fosab**	110.36	**0**	67.62	**0**	88.89	**0**	57.72	**0**
**egr1**	9.16	**0.001**	7.35	**0**	10.41	**0**	6.30	**0**
**egr2a**	21.42	**0**	44.20	**0**	17.28	**0**	7.98	**0**
**egr2b**	6.86	**0**	4.65	**0.002**	7.59	**0**	5.32	**0**
**egr4**	120.54	**0**	63.68	**0**	76.99	**0**	44.53	**0**
**jun**	4.17	**0**	4.57	**0**	9.38	**0**	5.34	**0**
**bdnf**	6.13	**0**	3.95	**0**	4.73	**0**	3.10	**0**
**eif4ebp2**	1.04	0.765	0.96	0.813	1.56	0.05	1.50	**0.01**

Supplementary Table 2 RT-qPCR values for IEG regulation in TL larvae treated with PTZ for one hour (+ PTZ) or treated with PROB 30min prior to the PTZ treatment (PROB + PTZ; **Figure 5i**). Expression values, respective to controls not treated with PTZ are >1 for upregulated IEGs and <1 for downregulated IEGs. *P*-values in bold = P < 0.05.

**Gene**	**TL (+ PTZ)**	*P*-value	**TL (PROB + PTZ)**	*P*-value
**fosab**	110.36	**0**	54.61	**0**
**egr1**	9.16	**0.001**	6.09	**0.001**
**egr2a**	21.42	**0**	39.38	**0**
**egr2b**	6.86	**0**	2.03	**0**
**egr4**	120.54	**0**	96.97	**0**

**jun**	4.17	**0**	1.44	0.073
**bdnf**	6.13	**0**	6.08	**0**
**eif4ebp2**	1.04	0.765	0.76	0.056

Supplementary Table 3 RT-qPCR values for gene regulation in PTZ treated (one hour) TL, DKO and PROB pretreated TL larvae compared to their respective controls (**Figure 7c**). Expression values are >1 for upregulated genes and <1 for downregulated genes. *P*-values in bold = P < 0.05. (TL+PROB group's control is treatment with PROB only)

**Genes**	**TL**	*P*-value	**DKO**	*P*-value	**TL + PROB**	*p*-value
kcnc3a	1.18	0.319	0.65	0.311	3.70	**0.001**
scn4bb	0.92	0.632	0.86	0.128	1.02	0.936
snap25b	1.01	0.946	0.83	0.346	1.02	0.9
sema6b	1.34	0.204	1.21	0.461	1.22	0.277
slc8a1b	0.97	0.895	0.87	0.302	1.73	**0**
slc8a2b	0.66	0.159	0.79	**0.029**	2.00	**0**
slco2b1	0.88	0.268	1.06	0.505	0.73	**0.008**
cacna1da	1.03	0.859	0.96	0.582	0.77	0.091
grin2bb	1.08	0.803	0.75	0.091	1.79	**0.02**
p2rx7	0.80	**0.004**	0.79	0.378	1.10	0.645
p2ry12	1.83	0.187	0.84	0.526	1.52	0.12
entpd1	1.71	**0**	1.32	**0.02**	1.29	0.07
nt5e	1.16	0.184	1.07	0.628	1.07	0.499
adora1b	1.02	0.959	1.42	0.133	0.65	0.331

Supplementary Table 4 Primers for RT-qPCR.

Gene	Gene Accession Number	Forward Primer (5'-3')	Reverse Primer (5'-3')
fosab	NM_205569	GTGAACGAAACAAGATGGCTG	TTTCATCCTCAAGCTGGTCAG
egr1	NM_131248	TCAACATATCCCAGTGCCAAG	TGTGTCTGGATGGGTTTCTG
egr2a	NM_001328404	CTTCTCCTGTGACTTCTGCG	GCTTTCTGTCCTTATGTCTCTGG
egr2b	NM_130997	GATGCGGAGAGGTCTATCAAG	AGGAGTAGGATGGCGGAG
egr4	NM_001114453	ACAGCACCTCAAAGACTACAG	ACGACAAGGTAAAAGACTGGAG
jun	NM_199987	CACAAGGCTCTGAAACACAAC	TGATGCCAGTTTGAGAAGTCC
bdnf	NM_001308648	ACAAGCGGCACTATAACTCG	ACTATCTGCCCCTCTTAATGG
eif4ebp2	NM_212803	AGTGACGGGCAAGAACATC	GTTGTTCACGTAGGTTCCTCTTC
kcnc3a	NM_001195240	CCATGATAGGGCCTGCTTC	AGAGATGTTATTGAGGCTGCG
scn4bb	NM_001077573	ACCTATGCCAGCTGTATTGG	CGCTCACGGTAAATTTGCAC
snap25b	NM_131434	TGAGAATTTGGAGCAGGTCG	TGTTGGAGTCAGCCATGTC
sema6ba	NM_001366315	TGATGGAGGGCTGTTTGTG	CGTTTGCGTGTTTGGGATC
slc8a1b	NM_001039144	GGAGGGACCAGTTTATTGAGG	GGCACGAAAGCAAAGAGAAC
slc8a2b	NM_001123284	TCACCAATGACCAGACAACTC	TGCACTCAACTGACCTTCTG
slco2b1	NM_001037678	AGATGGATTGGTGCTTGGTG	TTCTCAGTTGATGGCTCCAC
cacna1da	NM_203484	GGATGAGAAGGATAATGCCGAG	GGGTTTGTGTTGCTGAAGATG
grin2bb	NM_001128337	ATGAGGGACAGGGATAGAGG	AGGTTGGGATGAATGGGTTC
p2rx7	NM_198984	GTGTCATTTGTGGACGAGGAC	CACTCAACAGAGTCTTCATGCTG
p2ry12	NM_001308557	TCTTCGGTTTGATCAGCATCG	TCAGGATTACATTTGGGAGCG
entpd1	NM_001003545	ACCTGACCAACATGATTCCG	GCTGTTTTAGTAAAGCGACGG
nt5e	NM_200932	CAAACGGAAATGTGCTGGAG	GTCTGTCCCACTTGCTGAG
adora1b	NM_001128584	GGAACAATTTACACAGCCTGC	ACGAGCATGAAAAGCAGAGG
tuba1a (ref)	AF029250	GAGCGTCCTACTTACACCAAC	AGGGAAGTGGATACGAGGATAG
actb2 (ref)	NM_181601	GCCCCTAGCACAATGAAGATC	GACTCATCGTACTCCTGCTTG

Supplementary Movies

Supplementary Movie 1 PTZ-induced seizure-associated behavior stage II: rapid 'whirlpool-like' circling around the well.
'ZF_SZR_STII_circling_behavior.avi'

Supplementary Movie 2 PTZ-induced seizure-associated behavior stage III: convulsions, uncontrollable twitch of the body followed by a loss of posture and movement.
'ZF_SZR_STIII_convulsive_behavior.avi'
