# Supplementary material for: Panx1 channels promote both anti- and pro-seizure-like activities in the zebrafish via p2rx7 receptors and ATP signaling
Source: Commun Biol. 2022 May 18;5:472. doi: 10.1038/s42003-022-03356-2 (PMC9117279; doi:10.1038/s42003-022-03356-2)
Supplement: Supplementary file 2 — Description of Additional Supplementary Files [file 42003_2022_3356_MOESM2_ESM.pdf]

# Description of Additional Supplementary Files

**File name:** Supplementary Data 1
**Description:** Gene ontology enriched biological processes from FishEnrichR.

**File name:** Supplementary Data 2
**Description:** Clustergram genes and associated regulation values.

**File name:** Supplementary Data 3
**Description:** Data generated or analyzed during this study.

**File name:** Supplementary Movie 1
**Description:** PTZ-induced seizure-associated behavior stage II: rapid 'whirlpool-like' circling around the well. 'ZF_SZR_STII_circling_behavior.avi'

**File name:** Supplementary Movie 2
**Description:** PTZ-induced seizure-associated behavior stage III: convulsions, uncontrollable twitch of the body followed by a loss of posture and movement. 'ZF_SZR_STIII_convulsive_behavior.avi'
