# Supplementary material for: Panx1 channels promote both anti- and pro-seizure-like activities in the zebrafish via p2rx7 receptors and ATP signaling
Source: Commun Biol. 2022 May 18;5:472. doi: 10.1038/s42003-022-03356-2 (PMC9117279; doi:10.1038/s42003-022-03356-2)
Supplement: Supplementary file 9 — Reporting Summary [file 42003_2022_3356_MOESM9_ESM.pdf]

nature portfolio

Corresponding author(s): Georg R. Zoidl

Last updated by author(s): Apr 7, 2022

Reporting Summary

Nature Portfolio wishes to improve the reproducibility of the work that we publish. This form provides structure for consistency and transparency in reporting. For further information on Nature Portfolio policies, see our Editorial Policies and the Editorial Policy Checklist.

Statistics

For all statistical analyses, confirm that the following items are present in the figure legend, table legend, main text, or Methods section.

n/a	Confirmed	
☐	☒	The exact sample size (n) for each experimental group/condition, given as a discrete number and unit of measurement
☐	☒	A statement on whether measurements were taken from distinct samples or whether the same sample was measured repeatedly
☐	☒	The statistical test(s) used AND whether they are one- or two-sided<br>*Only common tests should be described solely by name; describe more complex techniques in the Methods section.*
☐	☒	A description of all covariates tested
☐	☒	A description of any assumptions or corrections, such as tests of normality and adjustment for multiple comparisons
☐	☒	A full description of the statistical parameters including central tendency (e.g. means) or other basic estimates (e.g. regression coefficient) AND variation (e.g. standard deviation) or associated estimates of uncertainty (e.g. confidence intervals)
☐	☒	For null hypothesis testing, the test statistic (e.g. F, t, r) with confidence intervals, effect sizes, degrees of freedom and P value noted<br>*Give P values as exact values whenever suitable.*
☒	☐	For Bayesian analysis, information on the choice of priors and Markov chain Monte Carlo settings
☒	☐	For hierarchical and complex designs, identification of the appropriate level for tests and full reporting of outcomes
☒	☐	Estimates of effect sizes (e.g. Cohen's d, Pearson's r), indicating how they were calculated

Our web collection on statistics for biologists contains articles on many of the points above.

Software and code

Policy information about availability of computer code

Data collection	Software used for data collection is cited in the manuscript and Supplementary Information.
Data analysis	Data analysis was completed using open source Matlab 2019b scripts, or other software (Graphpad(, as indicated.

For manuscripts utilizing custom algorithms or software that are central to the research but not yet described in published literature, software must be made available to editors and reviewers. We strongly encourage code deposition in a community repository (e.g. GitHub). See the Nature Portfolio guidelines for submitting code & software for further information.

Data

Policy information about availability of data

All manuscripts must include a data availability statement. This statement should provide the following information, where applicable:

- Accession codes, unique identifiers, or web links for publicly available datasets
- A description of any restrictions on data availability
- For clinical datasets or third party data, please ensure that the statement adheres to our policy

The datasets generated during and/or analyzed during the current study are available from the corresponding author on reasonable request

Field-specific reporting

Please select the one below that is the best fit for your research. If you are not sure, read the appropriate sections before making your selection.

☒ Life sciences ☐ Behavioural & social sciences ☐ Ecological, evolutionary & environmental sciences

For a reference copy of the document with all sections, see nature.com/documents/nr-reporting-summary-flat.pdf

Life sciences study design

All studies must disclose on these points even when the disclosure is negative.

Sample size	G*Power analysis was used to confirm the required sample size and the statistical power of the acquired data in line with what is published in Faul et al (Ref 73)
Data exclusions	No data were excluded from this study
Replication	All zebrafish larvae that were tested on within the same conditions (i.e. time of day, etc)were included in this manuscript. All larvae were reported on even if outcomes appeared to be outliers (i.e. panx1a-/- had 1 seizure-like event in the electrophysiological recordings and this was documented in the quantifications in figure 1)
Randomization	Randomization is not required as the testable variable is the genotype and the treatment with panx1 blocking pharmacology on the wild type population.
Blinding	For analysis purposes experimenters were kept blind to the genotypes or pharmacological treatments of larvae until data were processed.

Reporting for specific materials, systems and methods

We require information from authors about some types of materials, experimental systems and methods used in many studies. Here, indicate whether each material, system or method listed is relevant to your study. If you are not sure if a list item applies to your research, read the appropriate section before selecting a response.

Materials & experimental systems

n/a	Involved in the study	
☒	☐	Antibodies
☒	☐	Eukaryotic cell lines
☒	☐	Palaeontology and archaeology
☐	☒	Animals and other organisms
☒	☐	Human research participants
☒	☐	Clinical data
☒	☐	Dual use research of concern

Methods

n/a	Involved in the study	
☒	☐	ChIP-seq
☒	☐	Flow cytometry
☒	☐	MRI-based neuroimaging

Animals and other organisms

Policy information about studies involving animals; ARRIVE guidelines recommended for reporting animal research

Laboratory animals	Study involved use of danio rerio zebrafish larvae. Strain is Tubingen long fin (TL).
Wild animals	Study did not involve wild animals
Field-collected samples	Study did not involve samples collected from the field
Ethics oversight	All animal work was performed at York University’s zebrafish vivarium and in S2 biosafety laboratory in accordance with the Canadian Council for Animal Care guidelines after approval of the protocol by the Animal Care Committee (GZ: 2020-7-R3).

Note that full information on the approval of the study protocol must also be provided in the manuscript.
